# Y-box-binding protein 1 supports the early and late steps of HIV replication

**DOI:** 10.1371/journal.pone.0200080

**Published:** 2018-07-11

**Authors:** Caroline Weydert, Bart van Heertum, Lieve Dirix, Stéphanie De Houwer, Flore De Wit, Jan Mast, Steven J. Husson, Katrien Busschots, Renate König, Rik Gijsbers, Jan De Rijck, Zeger Debyser

**Affiliations:** 1 Division of Molecular Virology and Gene Therapy, Department of Pharmaceutical and Pharmacological Sciences, KU Leuven, Leuven, Belgium; 2 Laboratory for Photochemistry and Spectroscopy, Department of Chemistry, KU Leuven, Belgium; 3 Veterinary and Agrochemical Research Centre, VAR-CODA-CERVA, Brussels, Belgium; 4 Functional Genomics and Proteomics, Department of Biology, KU Leuven, 3000 Leuven, Belgium; 5 Systemic Physiological & Ecotoxicological Research (SPHERE), Department of Biology, University of Antwerp, 2000 Antwerp, Belgium; 6 Host-Pathogen-Interactions, Paul-Ehrlich-Institut, 63225 Langen, Germany; QIMR Berghofer Medical Research Institute, AUSTRALIA

## Abstract

The human immunodeficiency virus (HIV) depends on cellular proteins, so-called cofactors, to complete its replication cycle. In search for new therapeutic targets we identified the DNA and RNA binding protein Y-box-binding Protein 1 (YB-1) as a cofactor supporting early and late steps of HIV replication. YB-1 depletion resulted in a 10-fold decrease in HIV-1 replication in different cell lines. Dissection of the replication defects revealed that knockdown of YB-1 is associated with a 2- to 5-fold decrease in virion production due to interference with the viral RNA metabolism. Using single-round virus infection experiments we demonstrated that early HIV-1 replication also depends on the cellular YB-1 levels. More precisely, using quantitative PCR and an *in vivo* nuclear import assay with fluorescently labeled viral particles, we showed that YB-1 knockdown leads to a block between reverse transcription and nuclear import of HIV-1. Interaction studies revealed that YB-1 associates with integrase, although a direct interaction with HIV integrase could not be unambiguously proven. In conclusion, our results indicate that YB-1 affects multiple stages of HIV replication. Future research on the interaction between YB-1 and the virus will reveal whether this protein qualifies as a new antiviral target.

## Introduction

The human immunodeficiency virus (HIV) enters the cell through interaction with the cellular CD4 receptor and the CCR5 or CXCR4 co-receptors. After entry the viral RNA (vRNA) is reverse transcribed into double stranded viral DNA (vDNA). This nucleoprotein complex is referred to as pre-integration complex (PIC) and subsequently transported into the nucleus. In the nucleus, the PIC is targeted to the host chromatin where the viral integrase catalyses insertion of the viral genome into the host genome. After integration, the viral genome is silenced or transcribed into RNA which is exported in unspliced or spliced versions. These transcripts are translated by the cellular machinery into viral precursor proteins or used as novel RNA templates for packaging. These proteins are recruited for budding at the plasma membrane, leading to assembly and release of new particles. Upon cleavage of the viral polyproteins by the HIV-1 protease (PR) the mature virus is ready to infect new cells (for a review see [1]).

Like all viruses, replication of HIV depends on the interaction between viral and host proteins. For instance, viral entry is supported by the CD4, CCR5 and CXCR4 receptors, while nuclear transport, integration and RNA export are aided by proteins such as Transportin-SR2 (TRN-SR2, TNPO3), LEDGF/p75, and CRM1 respectively [2–8]. HIV cofactors are a promising source of new therapeutic targets as illustrated by the entry inhibitor maraviroc [9], a drug targeting the CCR5 co-receptor. LEDGINs are another example of promising antivirals in early clinical development targeting the LEDGF/p75 binding pocket on HIV-1 integrase (IN) [10,11].

Cellular proteins can also account for differences in host susceptibility to infection, as illustrated by the human cyclin dependent kinase p21 [12]. Experimental blockade of p21 leads to an increase in viral reverse transcripts and mRNA production. p21 is upregulated in a subset of CD4+ elite controllers, suggesting that it acts as a barrier against HIV infection. Finally, co-factors underlie distinct viral replication properties. Distinct integration site patterns observed between gammaretroviruses and lentiviruses, for instance, are determined by the use of different molecular tethers. The cellular bromodomain and extraterminal (BET) proteins interact with the gammaretroviral integrase, targeting integration towards enhancers and transcription start sites [13–15], while LEDGF/p75 interacts with lentiviral integrase targeting integration into the body of active genes [6,16].

**Fig 1:**
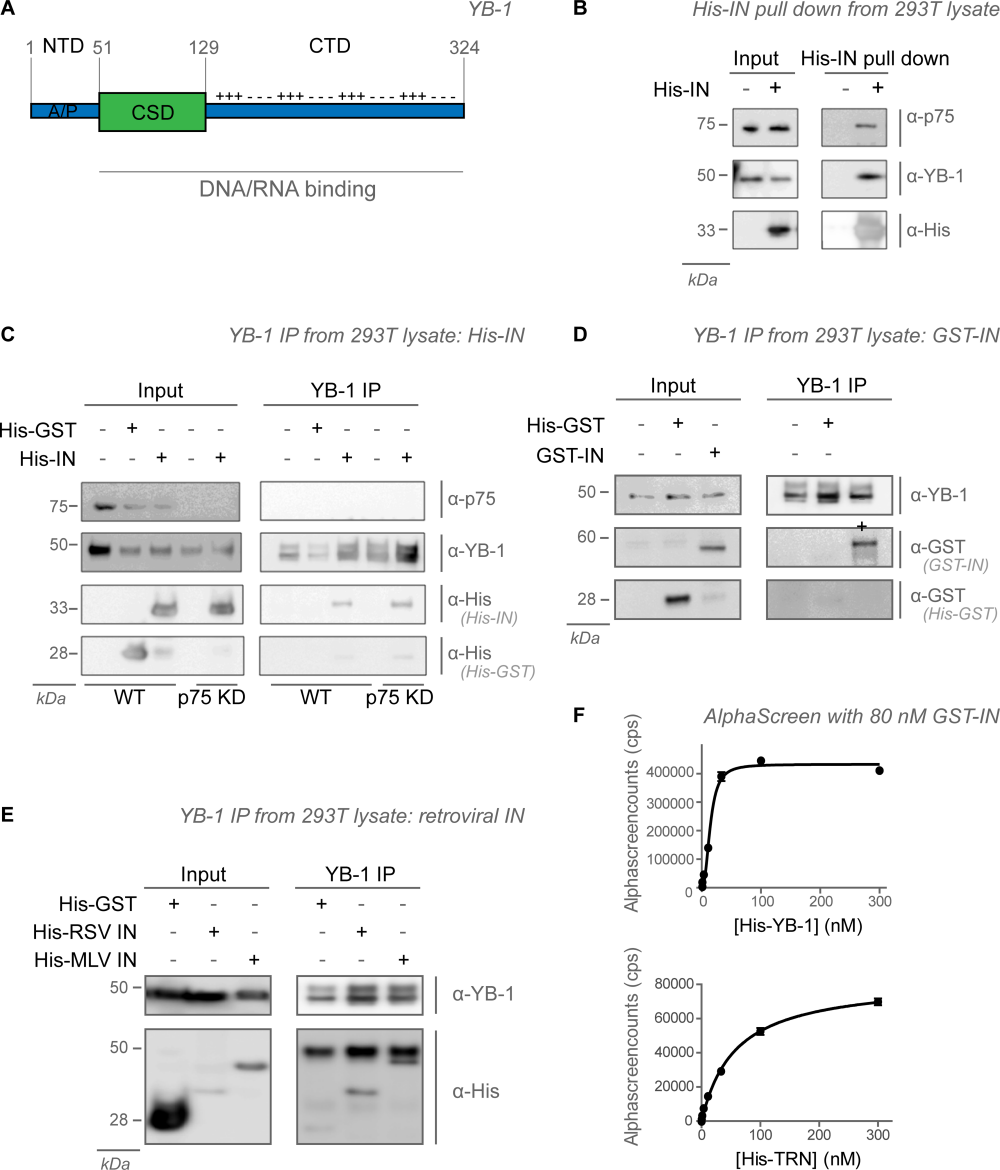
YB-1 is part of the integrase complex. (**A**) Schematic representation of YB-1. YB-1 contains an alanine/proline rich N-terminal domain (NTD), a conserved cold shock domain (CSD) and a C-terminal domain (CTD) that contains basic and acidic repeats. The CSD and CTD are responsible for the RNA and DNA binding properties. (**B**) Pull down of recombinant His-tagged HIV‑1 IN from spiked cell extracts of 293T cells. His-tagged protein, endogenous LEDGF/p75 and YB-1 were detected with specific antibodies after Western blotting. (**C**) Immunoprecipitation (IP) of endogenous YB-1 from 293T cell extracts in the presence or absence of LEDGF/p75 (p75 KD) spiked with recombinant His-IN and His-GST. His-tagged protein, endogenous LEDGF/p75 and YB-1 were detected with specific antibodies after Western blotting. (**D**) Co-IP of endogenous YB-1 with recombinant GST-His or GST-IN added to 293T cell lysates. The GST-tag and endogenous YB-1 were detected with specific antibodies after Western blotting. (**E**) IP of endogenous YB-1 from 293T extracts spiked with different His-tagged retroviral integrases and the His-GST negative control. All samples were analyzed by Western blot using the indicated antibodies. (**F**) Interaction of varying concentrations of recombinant YB-1 and 80 nM of IN as measured by AlphaScreen. The interaction between recombinant transportin-SR2 (TRN) and HIV-1 IN was analysed in parallel. Standard deviations for duplicates within one representative experiment are shown.

Here we report on the identification of Y-box-binding protein 1, or the nuclease-sensitive element-binding protein 1 (YB-1, YBX1 or NSEP1, from here on referred to as YB-1, uniprot accession code P67809), as a cellular cofactor of HIV replication. YB-1, a 324 amino-acid DNA and RNA binding protein, is involved in transcriptional and translational regulation, DNA repair and pre-mRNA splicing (recently reviewed by Lyabin et al. [17]). A murine YB-1 knockout (KO) resulted in disturbed neural tube formation, foetal growth retardation and prenatal death [18,19]. YB-1 contains an alanine/proline rich N-terminal domain (NTD), a highly conserved cold shock domain (CSD), and a highly charged C-terminal domain (CTD) that contains basic and acidic repeats (**Fig 1A**). YB-1 was initially named after its identification as a binder of inverted Y-box sequences in promotor regions [20,21]. However subsequent studies opposed these findings [22]. YB-1 displays high affinity for the RNA sugar-phosphate backbone (*K*_d_ = 10^-9^M) and a lower affinity for DNA (*K*_d_ = 10^-6^M) [23]. In the cytoplasm, YB-1 is a major component of messenger ribonucleoprotein complexes (mRNPs), with higher amounts in free mRNPs compared to polysomes. The YB-1/mRNA ratio is key for translational regulation, as both YB-1 levels close to saturation and complete YB-1 depletion inhibit mRNA translation [24,25]. YB-1 has been described to relocate into stress granules and into the nucleus upon stress related stimuli like a heat shock and UV-irradiation [26]. Similar results were obtained upon infection with mouse mammary-tumour virus (MMTV) and influenza virus, respectively [27,28].

YB-1 has been associated with a plethora of viruses. In the 1990’s YB-1 was found to bind to the promoters of Rous sarcoma virus (RSV), human T-cell lymphotropic virus-1 (HTLV-1), human neurotropic polyomavirus (JCV) and HIV-1, resulting in increased transcriptional activity upon overexpression [29–32]. Not much later YB-1 was shown to form a complex with the HIV Tat-TAR transcriptional complex [33]. In 2013, Mu *et al*. published that YB-1 enhances production of vesicular stomatitis virus glycoprotein G (VSV-G) pseudotyped HIV with a luciferase reporter gene (HIV_luc_) through the stabilization of newly produced vRNA [34]. Also murine leukaemia virus (MLV) vector production and MMTV production is regulated by YB-1 in an RNA-dependent manner [27,35]. Upon YB-1 overexpression, increased influenza and adenovirus production was observed while YB-1 was translocated to the nucleus [28]. On the other hand, depletion of YB-1 led to a 10–20 fold increase in Dengue virus production with only a 2-fold increase of translation [36]. Of note, in the latter study, YB-1 was completely depleted, while the other studies used a partial knockdown (KD). Also hepatitis C virus (HCV) replication is influenced by YB-1. HCV RNA replication is hampered by YB-1 depletion, while viral particle production is increased [37]. In summary, YB-1 is well known to affect late stage replication of several RNA viruses, presumably by interacting with vRNA. A recent report suggests that YB-1 may also interact with HIV-1 matrix [38].

The results presented in this manuscript reveal that YB-1, next to a role in late stage replication, also assists early replication steps of HIV. In consecutive experiments we show that i) YB-1 associates with integrase, ii) YB-1 is required for HIV replication, iii) YB-1 is required for late steps of HIV replication and iv) YB-1 plays a role in the early replication cycle at a stage between reverse transcription and nuclear import.

## Materials and methods

### Cell culture

HeLa TZM-bl (TZM-bl) cells were obtained through the NIH AIDS Reagent Program, Division of AIDS, NIAID, NIH: TZM-bl from Dr. John C. Kappes, Dr. Xiaoyun Wu and Tranzyme Inc [39]. HeLaP4 cells were a kind gift from Pierre Charneau, Institut Pasteur, Paris, France. SupT1 and HEK293T (293T) cells cells were obtained from the ATCC (Manassas, VA). HeLaP4, 293T and TZM-bl cells were maintained in DMEM glutamax^TM^ (GIBCO, Ghent, Belgium) supplemented with 5% fetal bovine serum (FBS, GIBCO, Ghent, Belgium) and 50 μg/ml gentamicin (GIBCO, Ghent, Belgium). SupT1 cells were maintained in RPMI 1640 (GIBCO, Ghent, Belgium) supplemented with 10% FBS and 50 μg/ml gentamicin. All cell lines were grown in a humidified atmosphere with 5% CO_2_ at 37°C.

### Plasmids

In order to generate stable KD cell lines, different miRNA-based short hairpin sequences (from here on referred to as miR) were cloned into the pGAE_sffv_BsdR_miRstemshuttle_WPRE plasmid, which was used for the production of Simian Immunodeficiency (SIV)-based viral vector particles (**S1A Fig**). First, pGAE SFFV-ZeoR-IRES-tCD34-2xshL3mir [40] was digested with *HindIII* and *SalI* and adaptors containing the miR30stemshuttle sequence (**S1 Table**, based on [41]) were ligated into the backbone, resulting in pGAE_sffv_ZeoR_miRstemshuttle_WPRE [42]. Secondly, a PCR amplified Blasticidine resistance cassette (BsdR) was introduced into this plasmid upon *AgeI* and *XhoI* digestion, primers in **S1 Table**, resulting in pGAE_sffv_BsdR_miRstemshuttle_wpre. Third, *LoxP* sequences were introduced using adaptor ligation (**S1 Table**) behind the SFFV promoter (*AgeI*/*BglII* digestion) and behind the wpre sequence (*Bsu36I* digestion). Lastly, the miRNA based short hairpin was cloned (**S2 Table**) upon *Esp3I* digestion. For YB-1 backcomplementation a synthetic codon optimized YB-1 sequence (YB-1s, IDT, Haasrode, Belgium) was PCR amplified, digested with *BglII* and *XhoI* and cloned into the pGAE_sffv_MCS_IRES_HygroR_WPRE backbone (**S1C Fig**).

In order to generate a construct for recombinant YB-1 expression, a synthetic codon optimized YB-1 cDNA was PCR amplified with primers containing the attB1 and attB2 sites (**S1 Table**) allowing the product to be cloned into pDONR221 (Invitrogen) using Gateway BP clonase. This reaction was followed by a Gateway LR clonase recombination reaction with pHGWA (for which we acknowledge Busso et al. [43]) resulting in pHGWA_YB-1s. All plasmids were sequence verified.

### Vector production

VSV-G pseudotyped SIV based lentiviral vectors were produced by transfection of 293T cells with 15 μg of transfer plasmid (pGAE_sffv_*LoxP*_BsdR_miR_WPRE_LoxP), 15 μg SIV packaging plasmid (pAd-SIV3+ (a kind gift from Didier Nègre, Ecole Normale Supérieure, Lyon)) and 5 μg pVSV-g per petridish as described before [44].

### Generation of stable cell lines

To deplete cells for YB-1, HeLaP4 or SupT1s were transduced with a dilution series of miRNA based viral vector and selected with Blasticidin (3–9μg/mL) (Invivogen, Toulouse, France). To restore YB-1 levels in YB-1 KD cell lines, two strategies were followed. The first strategy featured recombination of the *LoxP* sites after delivery of Cre recombinase to the KD with a similar construct containing no miR-resistant YB-1 (**S1D Fig**). The second strategy implied the use of an SIV-based lentiviral vector expressing miR-resistant YB-1 as described above (**S1C Fig**).

### Western blotting analysis and immunocytochemistry

For Western blotting, cells were lysed with 1% SDS. 10 μg cell lysate was separated by SDS-PAGE using 12.5% tris-glycine SDS-PAGE gels and transferred to a PVDF membrane. Membranes were probed with anti-YB-1 antibody (EP2708Y, 1:100000 or EP2706Y, 1:10 000, Abcam, Cambridge, UK), anti-β-actin antibody (AC15-A544, 1:50000, Sigma, Bornem, Belgium) or anti-LEDGF/p75 antibody (A300-847A, 1:500, ImTec, Antwerpen, Belgium). Detection was done with chemiluminescence using ECL (Pierce, ThermoScientific, Erembodegem, Belgium) after probing the blots with horseradish peroxidase coupled secondary antibody (1:1000–1:10 000, DAKO, Agilent technologies, Diegem, Belgium). Protein concentrations were determined using the Pierce bicinchoninic acid assay (BCA, Thermo Scientific, Erembodegem, Belgium).

For immunocytochemistry (ICC), 3 x 10^4^ HeLaP4 or 6 x 10^5^ SupT1 cells were seeded on PolyD-lysine coated chamber slides (Nunc, Roskilde, Denmark) and fixed with 2% paraformaldehyde 24 hours post seeding. YB-1 expression was detected with anti-YB-1 antibody (Abcam EP2708Y, 1:2000), followed by incubation with a Alexa488-conjugated secondary antibody (Life Technologies, ThermoScientific, Gent, Belgium) and DNA was stained with DAPI (1 μg/mL).

### Viral replication

One day prior to infection with 8 ng p24 HIV_IIIb_ or 190 ng p24 HIV_NL4.3_ in 2 mL DMEM, 2.1 x 10^4^ HeLaP4 cells were plated in a 6-well format. Cells were washed 24 hours post infection and capsid (p24) content of the medium was monitored daily. For viral replication experiments with suspension cells, 5 x 10^5^ cells were infected with 0.5–1 x 10^3^ ng HIV_NL4.3_ in 5 mL RPMI 1640 in T25 flasks and p24 production was followed over time. P24 production was measured by p24 ELISA (INNOTEST p24-ELISA (Innogenetics, Gent, Belgium).

### Nuclear import assay

The nuclear import assay has been performed as described before [45]. Briefly 3 x 10^4^ HeLaP4 cells were transduced with VSV-G pseudotyped HIV_luc_ containing IN-eGFP, provided with the Vpr-transcorporation technique (referred to as HIV_IN-eGFP_) [46]. 6 hours post transduction the cells were fixed with 4% paraformaldehyde and the nuclear lamina was stained with anti-lamin A/C antibody (Santa Cruz; sc-7292). Z-stacks were acquired with a Zeiss LSM510 multiphoton confocal microscope (Cell Imaging Core CIC, KU Leuven) and PICs were quantified with a MatLab routine described before [45].

### Analysis of infectivity/transduction efficiency

2 x 10^4^ HeLaP4 or 1 x 10^5^ SupT1 cells per well were plated in a 96-well plate and transduced with a dilution series of VSV-g pseudotyped HIV_luc_ (NIH AIDS reagent program), HIV_YFP_ (a kind gift from Dr. Mathias Lichterfeld [12], [47]) or an HIV-based lentiviral vector expressing eGFP and luciferase (LV_eGFP_t2A_fLUC) [48]. For HIV_luc_, transduction efficiency was determined through quantification of the luciferase activity (One Glo^TM^, Promega, Leiden, the Netherlands). Luciferase activity was normalized for protein content using a BCA assay (BCA Protein Assay Kit, Thermo Scientific, Ghent, Belgium). eGFP or YFP expression was determined by flow cytometry (Guava easyCite^TM^ 5HT Sampling Flow Cytometer, Merck Millipore) and analyzed using the InCite^TM^ software. 0.3 μM raltegravir (RAL), an IN strand transfer inhibitor, was included as a control. Data were plotted and analyzed with GraphPad Prism software.

### Analysis of HIV late effects

To quantify transcription and translation of the proviral DNA, 3 x 10^4^ HeLaP4 cells were plated in 96-well format one day prior to transfection using Lipofectamine 2000 (Life Technologies, Thermo Scientific, Ghent, Belgium) with 20–120 ng of plasmid encoding for HIV_luc_ or HIV_YFP_. 48 hours post transfection, cells were harvested and luciferase activity or mean fluorescence intensity was measured as described above.

To measure the effects on assembly and maturation, 6 x 10^5^ HeLaP4 cells were transfected with a HIV_NL4.3_ or HIV_YFP_ molecular clone. HIV capsid (p24) production was measured by the p24 ELISA (INNOTEST p24-ELISA (Innogenetics, Gent, Belgium)) and vRNA levels in cells or supernatant were measured as described before [49]. Briefly, RNA was extracted, reverse transcribed as described below and a quantitative PCR (qPCR) using the Gag primer-probe sets described in **S3 Table** was performed. Upon concentration using centrifugation over a cellulose membrane (Amplicon, Merck), Gag-processing in viral particles was verified with anti-CA antibody using Western Blotting analysis, as described above.

To measure the infectivity of viruses produced in the absence or presence of YB-1, TZM-bl cells were infected with supernatant containing an amount equal to 1 ng p24 of HIV_NL4.3_, 2 μM ritonavir (RTV), a protease inhibitor, was added 3 hours post infection. Cells were lysed 72 hours post infection with lysis buffer (50 mM Tris-HCl pH 7.3, 200 mM NaCl, 0.2% igepal, 5% glycerol), freeze thawed and analyzed for luciferase activity using ONE-Glo™ substrate (Promega, Leiden, the Netherlands).

### Quantitative PCR

To quantify YB-1 expression levels, RNA from 3 x 10^6^ cells was extracted using the Aurum^TM^Total RNA Mini Kit (Bio-Rad Laboratories, Nazareth, Belgium), followed by reverse transcription of 5 μg RNA into cDNA with the High Capacity cDNA Reverse Transcription Kit (Life Technologies, ThermoScientific, Gent, Belgium). YB-1 and β-actin transcripts were PCR amplified and detected using the primers and probes listed in **S3 Table** as described in [45].

In order to quantify viral intermediates, one day prior to infection 8 x 10^5^ HeLaP4 cells were seeded in a 6-well format. 4 hours post infection with 2.7 x 10^3^ ng p24 HIV_IIIB_ in 2 mL DMEM, cells were washed three times with PBS and the medium was replaced with DMEM containing 2 μM RTV, 0.3 μM RAL or 3 μM nevirapine (NVP, NIH AIDS reagent program). Samples were taken 4, 10, 24, 48 hours and 8–10 days post infection and genomic DNA was isolated using the Sigma Mammalian genomic DNA Miniprep kit (Sigma-Aldrich, Diegem, Belgium).

To quantify the integrated copy number from viral vectors and single round viruses (HIV_YFP_, HIV_luc_), HeLaP4 or SupT1 cells were transduced with viral vectors or single round virus as described above, transferred 3 days post infection to 6-well plates and harvested 8–10 days post infection. eGFP, YFP or Luc expression was analyzed, genomic DNA was extracted and PCR amplified with primers described in **S3 Table** and normalized for β-actin content.

All qPCR reactions contained 1 x IQ-supermix (Bio-Rad Laboratories, Nazareth, Belgium), 100–400 nM forward and reverse primer and 100–200 nM probe. For each experiment, a standard curve was generated and no-template controls were included. All qPCR experiments were run and analyzed in a Lightcycler®480 (Roche Life Science). Reverse transcripts (total HIV-1 DNA), 2-LTR circles and integrated copy number were measured with the primer sets in **S3 Table** and normalized for β-actin or RNAseP content.

### Integration site sequencing

As described above, cells were transduced with a dilution series of VSV-g pseudotyped LV_eGFP_t2A_fLUC vector, transferred to 6-well plates 2 days post transduction and kept in culture for 8–10 days. Genomic DNA was isolated and integration site distribution analysis was performed as described before [50].

### Transmission electron microscopy

HIV_NL4.3_ was produced in HeLaP4 cells in 6-well plates as described above. Cells were fixed 30 hours post transfection with 2.5% glutaraldehyde–2% formaldehyde in 0.1 M cacodylate buffer (pH 7.4) and postfixed in 1% OsO_4_. Ultra-thin sections were prepared as described before [51]. The samples were imaged in bright field (BF) mode using a Tecnai Spirit TEM (FEI, Eindhoven, The Netherlands) with Biotwin lens configuration operating at 120 kV. Micrographs were recorded using a 4*4 K CCD camera (Eagle, FEI) at various magnifications

### Cofactor identification by co-immunoprecipitation and mass spectrometry

Stable cell lines were prepared as described above, using a lentiviral vector encoding for HA-Flag-AU (HFA) codon optimized synthetic HIV integrase [52] (derived from pCG-HFA-HIV-IN, kindly provided by Dr. J. Skowronski, Cleveland, USA). Preparation of cellular and nuclear extracts, co-immunoprecipitation (co-IP), mass spectrometry (MS) and data analysis was performed as described earlier [13]. The top list of 25 cofactor hits were checked with co-IP and checked for inhibition of HIV_luc_ luciferase signal by specific siRNA’s as published by König *et al*. [53]. If 2 or more distinct siRNAs resulted in inhibited HIV replication of more than 30%, the cofactor was selected. The final prioritization was based on the co-IP ranking.

### Co-immunoprecipitation and pull down

1 x 10^7^ 293T cells were lysed in 150-CSK buffer (150 mM NaCl, 50 mM Tris pH 7.5, 0.8% NP-40) supplemented with complete protease inhibitor EDTA free (Roche Life Science) and DNaseI (3 units/mL, Roche Life Science). Cell lysates were allowed to stand on ice for 20 minutes, before they were centrifuged at 13000 g for 10 minutes. 100 nM of recombinant Glutathion-S-Transferase (GST)-tagged HIV-1 IN or His-tagged HIV-1 IN (His-IN) was added to the supernatant. The supernatant was pre-incubated for 1 hour with recombinant proteins and antibodies at 4°C prior to addition of 30 μL A/G+ agarose beads (Roche Life Science) or nickel NTA agarose (Qiagen, Antwerp, Belgium). After overnight incubation at 4°C, beads were washed 3 times with 550-CSK buffer (CSK buffer containing 550 mM NaCl). Bound proteins were detected by Western blot using anti-6xHis-tag Ab9108 (Abcam, Cambridge, UK), GST tag (Covance Research Products) or YB-1 (EP2708Y, Abcam, Cambridge, UK) specific antibodies.

### Protein purification and AlphaScreen binding assay

GST-IN and 6xHistidine-tagged Transportin-SR2 (His-TRN) were purified as described previously [54,55]. pHGWA_YB-1s was transformed in competent E. *coli* Rosetta cells. Briefly, cells were grown to an OD of 0.8 and protein production was induced with 0.5 mM Isopropyl β-D-1-thiogalactopyranoside and incubated for 4 h at 30°C. Cells were harvested, lysed and His-YB-1s was affinity purified over His-Select Nickel Affinity gel (Sigma), following the manufacturers’ instructions. DNAse and RNAse were added during all purification steps. Protein concentration was determined using the Pierce™ BCA Protein Assay Kit (ThermoScientific, Gent, Belgium).

In order to test direct binding between GST-IN and His-YB-1, we used the bead-based AlphaScreen protein-protein interaction technology (PerkinElmer) as described before [54]. Briefly, proteins were diluted to their respective working stocks in assay buffer (25 mM Tris/HCl pH 7.5, 150 mM NaCl, 1 mM dithiothreitol, 1 mM MgCl2 0.1% (w/v) BSA, 0.1% (v/v) Tween 20). 10 μL of His-YB-1 or His-TRN-SR2 and 5 μL of GST-IN were pipetted in a 384-well OptiPlate (PerkinElmer), mixed and incubated at 4°C for 1 hour. Next, 10 μL of glutathione donor and Ni-chelate acceptor AlphaScreen beads (20 μg/ml final concentration each) were added and the plate was incubated for 1 hour at 30°C. The plate was read in an EnVision Multilabel plate reader (PerkinElmer) and the AlphaScreen signal data were analyzed using GraphPad Prism 5.0 software. Whereas GST-IN was kept constant at 80 nM, His-YB-1 and His-TRN were titrated in a 1:3 dilution series starting at 300 μM.

## Results

### YB-1 interacts with retroviral integrases

To identify new HIV integrase interacting proteins we generated a stable 293T cell line overexpressing double tagged HIV IN (Flag-HA-IN). After immunoprecipitation of Flag-HA-IN, co-immunoprecipitating proteins were analysed by nanoLC-Q-Tof MS [13]. To identify proteins with a potential role in HIV replication, hits were cross-referenced with a genome wide siRNA screen [53], resulting into a shortlist of potential co-factors. The list including the respective Mass Spectrometry analysis after immunoprecipitation (MS) and siRNA scores is depicted in **S4A Table**. Based on their ranking and cellular function, the effect on HIV transduction efficiency 3 of these factors was re-evaluated by siRNA and miRNA-mediated depletion (**S4B Table**). This parallel and comparative analysis revealed that YB-1 had the most pronounced impact on HIV replication.

To confirm that YB-1 is found in a complex with HIV-1 IN, 293T cell lysates were spiked with recombinant His-IN. Upon His-IN pull down, endogenous YB-1 (**Fig 1B**) was readily detected, while YB-1 could not be detected in the absence of His-IN. The detection of LEDGF/p75, a well characterized binding partner of HIV-1 IN, validates the assay [6]. In a reciprocal experiment, we were able to detect spiked His-tagged IN after immunoprecipitating endogenous YB-1, but not His-Glutathione-S-transferase (His-GST) fusion protein (**Fig 1C)**. Similar results were obtained using GST-tagged IN (GST-IN, **Fig 1D**). To study the viral specificity, YB-1 was immunoprecipitated from 293T cellular extracts spiked with His-tagged IN from RSV or MLV (**Fig 1E**). RSV and MLV IN were detected upon YB-1 precipitation, while the His-GST negative control was not. These results suggest that YB-1 can be a part of retroviral IN complexes. We detected a direct interaction between YB-1 and HIV-1 IN *in vitro*, using recombinant, purified His-tagged YB-1 (His-YB-1) and GST-IN in an AlphaScreen binding assay (**Fig 1F**). Binding of His-tagged Transportin-SR2 (His-TRN) to GST-IN was included as a positive control (**Fig 1F**) [56]. Although we were able to detect a direct interaction between recombinant YB-1 and IN, YB-1 bound to other non-related proteins (Menin, Cell division cycle-associated 7-like protein (CDCA7L/JPO2), LEDGF/p75, Transportin-SR2, **S2 Fig**). As such, direct binding between YB-1 and IN could not be unambiguously shown.

### YB-1 supports HIV replication

**Fig 2:**
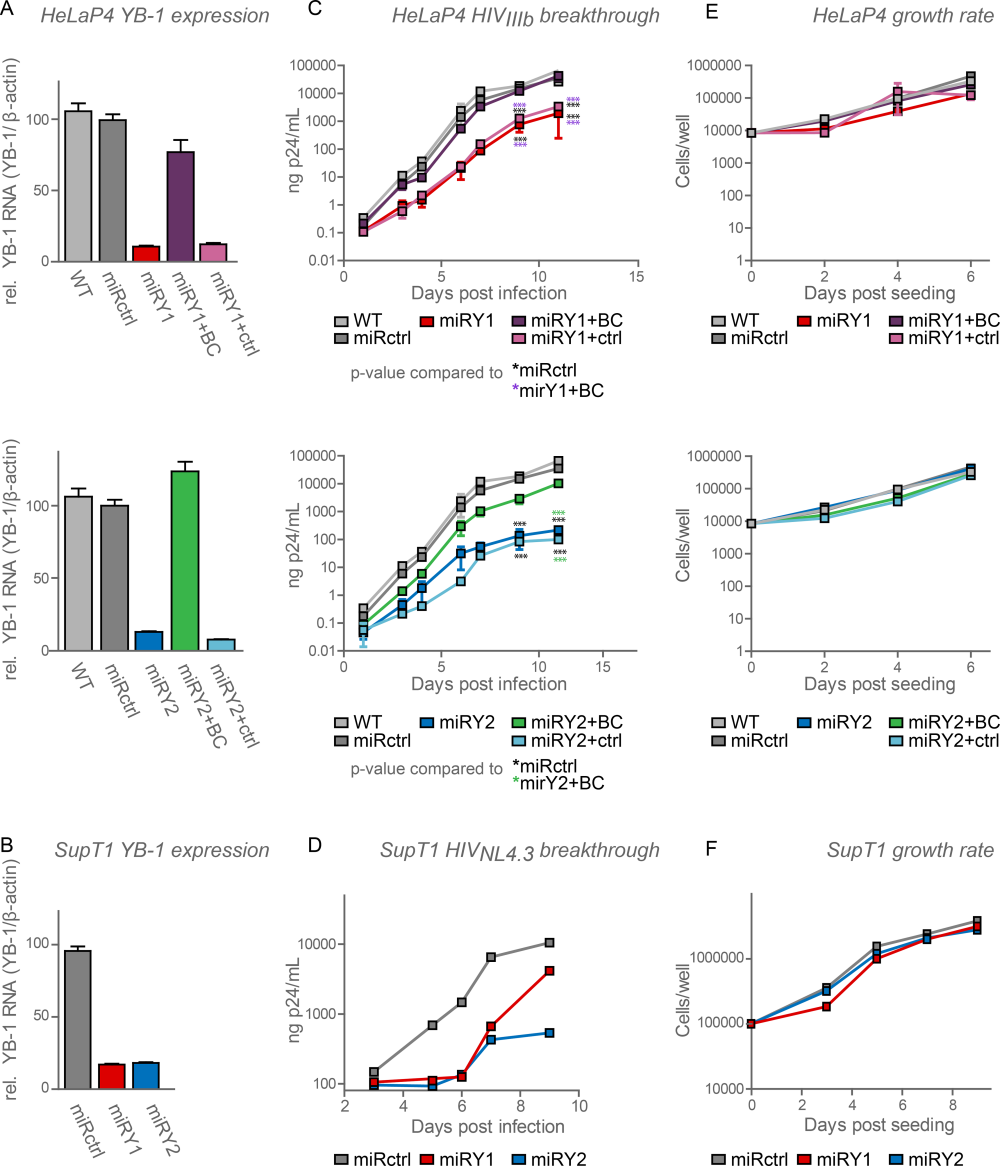
YB-1 supports HIV replication. Replication of HIV_IIIB_ in HeLaP4 and HIV_NL4,3_ in SupT1 cells stably expressing miRNA-based shRNAs against DS Red (negative control, miRctrl, *grey*) or YB-1 (miRY1, *red*, miRY2, *dark blue*). As a control for aspecific targeting, YB-1 levels were backcomplemented (miRY1+BC, *purple*, miRY2+BC, *green*). BC was also controlled with an empty cassette vector (miRY1+ctrl, *pink*, miRY2+ctrl, *light blue*) in HelaP4 cells. (**A**, **B**) Levels of YB-1 mRNA as determined with RT-qPCR in (**A**) HeLaP4 and (**B**) SupT1-derived cell lines. Standard deviations for triplicates within one experiment are shown. (**C**, **D**) Upon HIV infection capsid production in the supernatant of the different cell lines was followed over time using p24-ELISA as a measure for HIV replication. Standard deviations for triplicate data points are shown in (**C**). (**A-D**) Shown are representative experiments out of minimum 3 experiments. (**E**, **F**) As a measure of cell proliferation, cell growth of uninfected (**E**) HeLaP4 and (**F**) SupT1-derived cell lines was monitored daily in parallel with the breakthrough experiments. Error bars indicate standard deviations of duplicate data points. Differences in panel C were determined using two-way ANOVA, followed by the Bonferroni multiple comparison test. The YB-1 depleted cell lines were compared to the miRctrl (*black stars*) and the BC conditions (*purple* and *green stars* for miRY1+BC and miRY2+BC, respectively). *p < 0.*05*, **p < 0.*01*, ***p < 0.*001*.

In order to investigate a potential role of YB-1 during lentiviral replication, we depleted the protein stably using two micro-RNA based short-hairpins (from here on referred to as miR) in HeLaP4 and SupT1 cell lines (miRY1 and miRY2). A miR against DsRed was used as a negative control (miRctrl). YB-1 levels were restored upon transduction of the knockdown cell lines with a SIV-based lentiviral vector expressing miR-resistant YB-1 (BC: backcomplementation: miRY1+BC and miRY2+BC). Cells transduced with the same vector, without YB-1 backcomplementation (miRY1+ctrl and miRY2+ctrl) were used as controls for antibiotic selection and vector transduction. Levels of YB-1 expression were verified by RT-qPCR (**Fig 2A and 2B**). MiRY1 and miRY2 depleted YB-1 by 90% and more than 85%, respectively, in HeLaP4 cells and more than 80% in SupT1 cells. Viral replication of HIV_IIIB_ in the HeLaP4-derived cell lines was followed over time revealing that YB-1 depletion resulted in a 10-fold reduction of viral replication (**Fig 2C**)**.** Similar results were obtained for HIV_NL4.3_ (data not shown) and in T-cell-derived SupT1 cells (**Fig 2D**) for both miRs (**Fig 2C and 2D**). The phenotype could be rescued upon restoration of the YB-1 levels (**Fig 2C**). Distinct proliferation rates between the different cell lines were not responsible for the observed phenotype, as no major growth deficits were observed when depleting YB-1 (**Fig 2E and 2F**). All together these data show that YB-1 supports efficient HIV replication.

### Viral particle production and viral RNA levels are reduced upon YB-1 depletion

**Fig 3:**
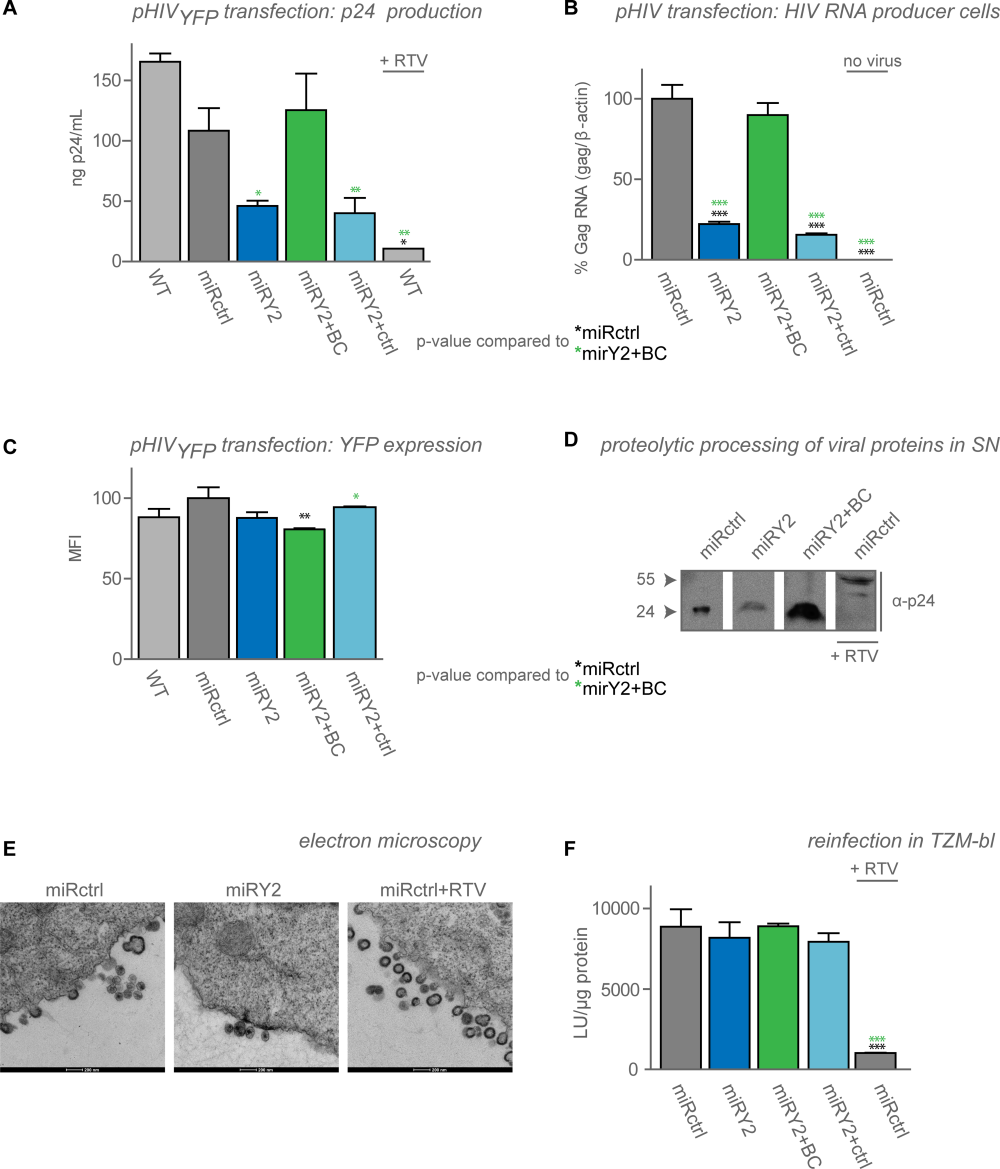
Viral RNA levels and viral particle production are hampered by YB-1 depletion. Transfection of HeLaP4 cells with plasmid encoding for HIV_NL4.3_ (pHIV) or HIV_YFP_ (pHIV_YFP_) resulted in (**A**) lower capsid (p24) production as measured with p24 ELISA and (**B**) lower total viral Gag RNA levels as measured with qPCR when YB-1 was depleted (miRY2, *dark blue*) compared to control cell lines (WT, *light grey*, miRctrl, *dark grey*). 2 μM Ritonavir (RTV) was added as control in (**A**). Both capsid and viral RNA levels were rescued when the YB-1 levels were restored (miRY2+BC, *green*), but not when the cells were transduced with a control vector (miRY2+ctrl, *light blue*). (**C**) Transfection of HeLaP4-derived cell lines with HIV_YFP_ (pHIV_YFP_). YFP expression levels were measured by flow cytometry. (**D**) Western blot of concentrated supernatant (SN) from virus producing cells with anti-capsid (p24) antibody. (**E**) TEM images of sections from virus producing cells using negative staining 30 hours post transfection. (**F**) Infectivity of virus produced in the presence or absence of YB-1 was determined in TZM-bl reporter cells. Shown are luciferase units (LU) per μg protein. (**A**, **C**, **D**, **F**) Representative experiments out of minimum 3 independent experiments are shown. Error bars indicate standard deviations of (**A**) duplicate or (**B-D, F**) triplicate data points. Statistical differences were determined using one-way ANOVA, followed by the Bonferroni multiple comparison test. The YB-1-depleted cell lines were compared to the miRctrl (*black stars*) and the BC conditions (*green stars*). *p <0.*05*,**p < 0.*01*, ***p < 0.*001*.

**Fig 4:**
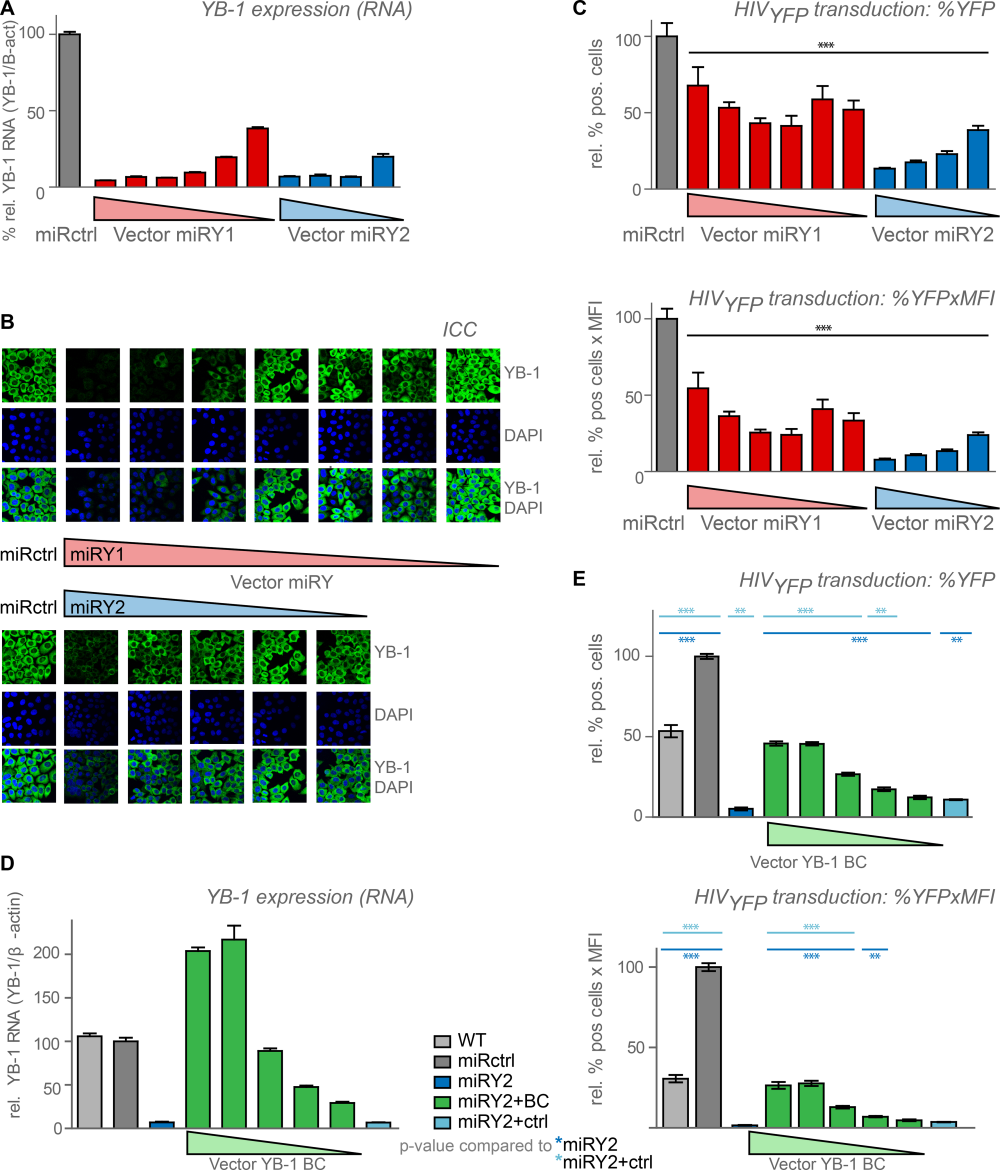
YB-1 levels affect the early steps of HIV replication. HeLaP4 cells were transduced with a dilution series of vector expressing a miRNA-based shRNA against YB-1 (miRY1, *red*, miRY2, *blue*) or against DSRed (miRctrl, *dark grey*). YB-1 expression levels were measured with (**A**) RT-qPCR and with (**B**) immunocytochemistry (ICC). YB-1 was detected with a specific antibody (*green*). DNA was stained using DAPI. (**C**) The stable cell lines were infected with VSV-G pseudotyped HIV_YFP_ and harvested 48–72 hours post infection. % YFP positive cells (*upper panel*) and the % YFP positive x mean fluorescence intensity (MFI) (*lower panel*) were measured using flow cytometry. Shown is a representative experiment out of 3 independent experiments. Standard deviations for triplicates within one experiment are shown. The various YB-1-depleted cell lines were compared to the miRctrl (*black stars*) condition using one way ANOVA followed by the Bonferroni multiple comparison test. (**D**) HeLaP4 cells depleted for YB-1 were transduced with different concentrations of vector expressing miRNA-resistant YB-1 (miRY2+BC, *green*) or control vector (miRY2+ctrl, *light blue*) and RNA expression levels were determined with RT-qPCR. (**E**) Experiment performed as in panel C. VSV-G pseudotyped HIV_YFP_ infection efficiency was partially restored upon YB-1 backcomplementation, as measured by flow cytometry. Shown is a representative experiment out of 3 independent experiments. Standard deviations of triplicate data points are shown. Differences were determined using one-way ANOVA, followed by the Bonferroni multiple comparison test. The miRctrl and the BC dilution series were compared to the miRY2 (*dark blue stars*) and miRY2+ctrl (*light blue stars*) conditions. *p <0.*05*,**p < 0.*01*, ***p < 0.*001*.

YB-1 plays a role in late steps of replication of several RNA viruses [27,34,35,37]. To evaluate the role of YB-1 during late steps of HIV replication we transfected the HeLaP4-derived cell lines with plasmid encoding HIV_NL4.3_ or HIV_YFP_ and monitored p24 production in the supernatant (**S3A and S3B Fig** for miRY1, **Fig 3A and S2B Fig** for miRY2) as a measure for late steps until proteolytic maturation. HIV_YFP_ is a single round virus defective for *env* which expresses a yellow fluorescent protein (YFP) reporter [12,47]. This YFP sequence is cloned between env and nef, using an internal ribosome entry site (IRES). Depletion of YB-1 in these cell lines resulted in a 2- to 5- fold decrease in p24 production which was rescued after restoration of YB-1 levels. Similarly, the relative amount of cellular vRNA levels (Gag/β-actin) were 2–5 fold lower when YB-1 was depleted (**Fig 3B and S3C Fig**). These results are in accordance with a previous study claiming that reduced viral production is due to impaired RNA stabilization [34]. However, lower RNA levels did not consistently result in lower YFP protein levels; YFP expression remained similar (**Fig 3C and S3D and S3E Fig**) or decreased maximum 2-fold (**S3F Fig**). This effect was not due to overexpression of YFP, since transfection with different amounts of plasmid resulted in a similar phenotype (**S3D Fig compared to Fig 3C**). Neither was the effect due to YB-1 expression levels, as transduction with different amounts of the YB-1 backcomplementation vector did not result in altered YFP expression (**S3E Fig,** YB-1 expression in **Fig 4D**). Transfection of pHIV_luc_ resulted in similar results. Yet, YFP or luciferase are encoded in the Nef gene and hence are considered Rev-independent (while gag vRNA export is dependent on rev). Furthermore, the MFI might not be sensitive enough to robustly measure differences. Additionally, we analyzed the Gag/Gag-Pol expression levels in a semi-quantitative manner by Western blotting analysis (**S4 Fig**), showing an approximately 5-fold lower reduction of gag p55 expression in case of YB-1 depletion. Gag p55 levels partially recovered when YB-1 levels were restored. Furthermore, we could not detect defects in Gag proteolytic processing in virus produced in YB-1 KD cell lines (**Fig 3D**) although proteolytic cleavage of Gag remained sensitive to ritonavir (RTV) inhibition, as measured with Western Blotting (**Fig 3D**). No detectable defects in maturation or budding were observed by transmission electron microscopy (TEM, **Fig 3E**). The virus produced in YB-1 depleted cells, normalized on p24-content, showed no defects in infectivity when tested on TZM-bl cells, which contain a luciferase reporter gene under control of an LTR promotor (**Fig 3F**). All together these data indicate that YB-1 is required for maintaining the vRNA level and efficient viral particle production.

### YB-1 is important for the early steps of HIV replication

To examine whether early steps in the HIV replication cycle were affected by YB-1 depletion, we transduced the YB-1 KD cells with a VSV-G pseudotyped single round virus (HIV_YFP_). As initial results revealed that a drop in transduction efficiency did not consistently correlate with YB-1 KD, we hypothesized that our this transduction deficiency might be strictly dependent on the actual YB-1 levels. In order to address this hypothesis, we transduced HeLaP4 cells with a 1:3 dilution series of the SIV-based vector expressing miRY1 or miRY2, resulting in varying YB-1 expression levels. Differential knockdown was confirmed by RT-qPCR and confocal microscopy (**Fig 4A and 4B**). Transduction of these YB-1 knock-down cell lines with different MOIs of HIV_YFP_ resulted in an approximately 2- and 5-fold decrease in YFP positive cells with miRY1 and miRY2, respectively (**Fig 4C and S5A and S5B Fig**). Surprisingly, strong knock-down of YB-1 (qPCR >95%, resulting in undetectable levels of YB-1 in ICC) reversed the effect on transduction efficiency (**Fig 4A, 4B and 4C and S5A and S5B Fig**). These results support a concentration-dependent role for YB-1 during the early steps of HIV replication. Off-target effects were less likely since the percentage of transduced cells could be rescued when YB-1 levels were restored (miRY1+BC, mirY2+BC), but not in the control condition (miRY1+ctrl, miRY2+ctrl) (**Fig 4D and 4E and S5C, S5D and S5E Fig)**. Again, the percentage YFP-positive cells was shown to correlate with the YB-1 expression levels (**Fig 4D and 4E and S5C and S5D Fig**).

**Fig 5:**
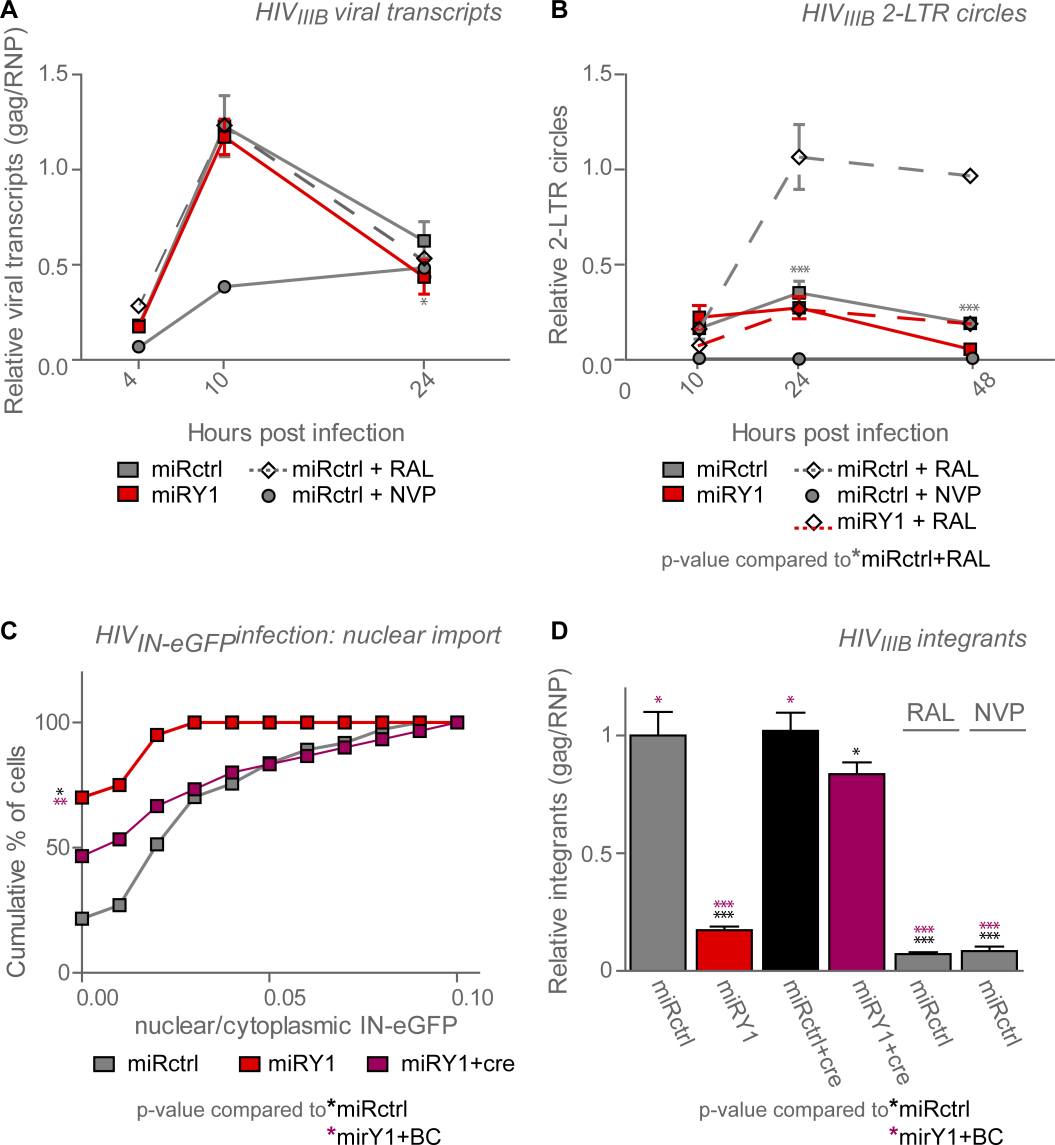
YB-1 depletion inhibits a step between reverse transcription and nuclear import. HeLaP4 YB-1 knock-down (miRY1, *red*) or control (miRctrl, *dark grey*) cells were infected with HIV_IIIB_ in the presence of 2 μM ritonavir (RTV) and the indicated compounds (0.3 μM raltegravir (RAL), 3 μM nevirapine (NVP)). Cells were sampled at different time points, genomic DNA was extracted and qPCR was performed for HIV-1 (**A**) Gag and (**B**) 2-LTR circles to monitor reverse transcription and nuclear import, respectively. (**C**) HeLaP4 YB-1 knock-down (miRY1, *red*) or control (miRctrl, *dark grey*) cells were infected with fluorescent HIV_IN-eGFP_ and fixed 5 hours post transduction. As a backcomplementation control the miRY1 was floxed out using Cre–recombinase (miRY1+cre). Green fluorescent particles in the cytoplasm and nucleus were quantified by laser-scanning confocal microscopy. The cumulative distribution of the percentage of cells containing a certain ratio of fluorescent nuclear over cytoplasmatic IN-eGFP particles is depicted. One out of two representative experiments is shown. (**D**) Cells as described for (**A**) and (**B**) were sampled 10 days post infection and a qPCR for Gag DNA was performed as a measure for integration. Standard deviations of triplicate data points are shown. (**A**, **B**, **D**) qPCR data were normalised for RNaseP (RNP). Differences were determined using (**A**, **B**) two-way ANOVA, (**C**) Mann Whitney test or (**D**) one-way ANOVA, followed by the Bonferroni multiple comparison test. Statistical differences between (**A**) miRY1 and miRctrl and (**B**) miRY1 +RAL and miRctrl+RAL are shown. (**C**, **D**) Cell lines were compared to the miRctrl (*black stars*) and miRY1+cre (*violet stars*) conditions. *p <0.*05*,**p < 0.*01*, ***p < 0.*001*.

To investigate the steps in early replication affected by YB-1, we infected the HeLaP4-derived cell lines with HIV_IIIB_, and quantified viral intermediates at different time points. To inhibit reinfection 2 μM RTV was added 4 hours post infection. Controls using 3 μM nevirapine (NVP) as an inhibitor of reverse transcription and 0.3 μM raltegravir (RAL) as an integrase strand transfer inhibitor were included. Reverse transcripts were not affected by YB-1 KD (**Fig 5A**). 2-LTR circles are dead-end by-products of viral replication, and are only formed in the nucleus. Therefore they can be used as indirect measurement for nuclear import. It was shown before that due to the block of integration upon addition of integrase strand transfer inhibitors the number of 2-LTR circles drastically increases **Fig 5B** [57]. However, under the same conditions YB-1 KD did not result in an increased formation of 2-LTR circles (**Fig 5B**), pointing towards a defect in nuclear import. A more direct way to measure HIV nuclear import makes use of IN-eGFP labelled viral particles [45]. Therefore HeLaP4 YB-1 knockdown or control cells were infected with a VSV-G pseudotyped virus carrying green fluorescently labelled IN (HIV_IN-eGFP_) incorporated in the particle through Vpr-trans-incorporation, as published before [45]. The ratio of nuclear versus cytoplasmic fluorescent IN-eGFPs measures the efficiency of the nuclear import process. The IN-eGFP ratio was clearly reduced after YB-1 depletion whereas partial rescue was obtained upon Cre-mediated removal of the knockdown cassette (*miRY1 + cre*) (**Fig 5C and S6A Fig**). This lower number of nuclear green fluorescent integrase complexes was not a consequence of impaired cellular entry or PIC stability, as the number of fluorescent integrase complexes per cell did not significantly differ between the cell lines (**S6B Fig**), nor was there a significant increase of these complexes at the nuclear membrane upon YB-1 depletion (**S6C Fig**). The block in nuclear import was reflected in the lower number of integrated copies upon YB-1 depletion which could be rescued upon restoration of the YB-1 knockdown levels (**Fig 5D**). These results suggest that YB-1 depletion blocks a step in between reverse transcription and nuclear import. Finally, to test the integration site distribution, HeLaP4 cells were transduced with a lentiviral vector encoding eGFP and luciferase. Transduction efficiency and integrated copies were followed with flow-cytometry and qPCR, but no significant changes in HIV integration site distribution were observed upon YB-1 KD (**S5 Table**).

## Discussion

Y-box-binding protein 1 (YB-1), a multifunctional RNA and DNA binding protein, is involved in the replication cycle of many viruses [27,28,31,35–37,58]. Here we reveal that YB-1 is necessary for efficient HIV-1 replication (**Fig 2**). Using a highly controlled RNAi-based YB-1 knockdown approach in both HeLaP4 and SupT1 cells, we show that YB-1 depletion reduces spreading HIV-1 replication 10-fold due to early and late step replication deficits.

We used a proteomics approach (Co-IP + MS analysis) to identify new binding partners of HIV-1 integrase (**S4 Table**). Next to the known HIV integrase-interactor LEDGF/p75, YB-1 appeared among the top candidates in the HIV IN screen (**S4 Table**). Although we confirmed the presence of YB-1 in the HIV IN complex through pull-down and co-IP (**Fig 1B**–**1E**), attempts to identify a direct interaction were inconclusive due to non-specific interactions observed in recombinant protein samples (**Fig 1F**). Non-specific interactions could be induced by the high affinity of YB-1 for contaminating RNA and DNA. However, attempts to reduce DNA and RNA levels through the addition of nucleases did not eliminate the observed interactions. Likewise, the high affinity of YB-1 for DNA and RNA and the specific unstructured nature of YB-1 [17] hampered the delineation of YB-1 domains responsible for the interaction. YB-1 has been detected in various other screens for HIV cofactors [38,53,59]. In the König screen, an siRNA screen combined with interrogation of human interactome databases, YB-1 was classified as part of the Gag interactome [53]. In the Jäger screen, a large scale co-IP for viral proteins, YB-1 appeared as a hit for all HIV proteins, suggesting that YB-1 may have been a non-specific binder in that particular screen [59]. The most recent proteomics screen, performed by Li *et al*., claims that YB-1 was interacting with the HIV-1 matrix protein [38]. We originally detected YB-1 as an interactor of not only HIV-1 IN but also MLV IN and RSV IN in parallel proteomic screens. Still, in each instance the interaction was only detected in cytosolic extracts, not in nuclear ones and not in control cell lysates lacking retroviral IN. Although we cannot make firm conclusions on any direct interaction with a specific HIV protein, a phenotypic impact on HIV replication upon depletion of YB-1 is clearly documented (**Fig 2**).

Consistent with Mu *et al*., who demonstrated that YB-1 overexpression results in an enhanced viral vector particle production and a stabilization of newly produced vRNA through binding of stem loop 2 [34], we show here that YB-1 depletion reduces cellular HIV-1 Gag RNA levels and viral p24 production (**Fig 3A and 3B**). The specific drop in vRNA could be explained by reduced transcription from the HIV LTR or decreased RNA stability. In fact, Ansari *et al*. demonstrated that YB-1 is able to bind Tat *in vitro* and that it can influence the expression of a reporter gene under control of a minimal HIV promotor [33]. The fact that expression of YFP or luciferase from the *nef* locus was not sensitive to YB-1 depletion excludes a general LTR defect in our experiments. We tend to agree with Mu *et al*. that YB-1 probably stabilizes genomic vRNA without affecting transcription per se. This hypothesis is in line with the fact that we could not detect YB-1 relocalization to the nucleus upon HIV-1 infection or transfection in HeLaP4, which would be expected if YB-1 would affect LTR-mediated transcription. Moreover, in co-IP experiments (**S4 Table**) the IN/YB-1 complex was detected in the cytosol, not in the nucleus. The fact that YB-1 is important for RNA metabolism, in particular as a major component of free mRNPs and polysomes (recently reviewed by [17]), supports the RNA stabilization hypothesis. Finally, YB-1 has been described to support viral particle production of MLV, Influenza, HCV and MMTV in an RNA-dependent manner [27,28,35,37]. In case of HCV, depletion of YB-1 resulted in a decrease in vRNA, which was not translated into a decreased HCV protein production but resulted in a lower infectivity in the next round [37]. Paranjape *et al*. on the other hand demonstrated that YB-1 is a translational repressor for Dengue Virus production [36]. These at first sight opposite effects, could be explained by the fact that Paranjape *et al*. used YB-1 KO cells for their research, in contrast to all other groups which used a KD or overexpression approach. As it was previously published that the YB-1/mRNA ratio determines if translation is either promoted or blocked, this complete lack of YB-1 may explain the paradoxical phenotype (recently reviewed by [17]). A complementary explanation implies that YB-1 KD leads to a rev-dependent defect, and subsequently lower levels of gag vRNA, but not of the genes expressed in the nef locus. Hence, more research is necessary to explore this hypothesis.

Whereas late effects of YB-1 on RNA virus replication have been documented before, we here show for the first time that YB-1 depletion also hampers HIV-replication at a step between reverse transcription and nuclear import, resulting in a drop in nuclear GFP-integrase complexes and integrated copy number (**Fig 5**). Of note, transduction efficiency of an MLV-derived vector was not severely hampered by YB-1 KD suggesting that the early effect is specific (**S7 Fig**). The block in transduction efficiency was dependent on YB-1 levels (**Fig 4**). Near full depletion of YB-1 apparently reduced the replication deficit hinting at multiple dose-dependent mechanisms. Nonetheless, other mechanisms cannot be excluded. Our data also revealed that YB-1 depletion does not affect integration site distribution (**S5 Table**). HIV nuclear import-factors like Transportin–SR2 and RanBP2 are known to influence integration site distribution to some extent, suggesting that YB-1 affects HIV replication via a different mechanism [60].

The HIV and MLV nuclear import mechanisms are different. While MLV is believed to require mitosis to reach the chromatin [61,62], HIV can enter the nucleus through the nuclear pores [63]. Several viral features might account for these differences including uncoating [64], vDNA conformation/compacting [65,66] and the usage of specific nuclear import/docking receptors [63,67]. Since YB-1 is an RNA/DNA binding protein it is tempting to speculate that YB-1 influences vDNA conformation/compacting required for HIV nuclear import. Following this theory, an optimum YB-1 concentration may be required for correct compaction of nucleic acids. An alternative hypothesis implies that another host factor functionally compensates very low YB-1 levels and restores transduction efficiency. In general, the observed early effect is complex, might be caused by multiple factors and requires further study. HIV co-factor identification and validation has paved the way to antiviral drug discovery successes during recent years [68–71]. Although we show that YB-1 is an important HIV-1 co-factor, targeting YB-1 or its interactions with viral proteins may not be straightforward. Its RNA interacting properties, its ‘Goldilocks effect’ on HIV replication, as well as the lack of strong evidence for a direct interaction with a particular HIV protein jeopardize successful drug development. However, as high YB-1 levels have been associated with poor prognosis and disease recurrence in several human malignancies (recently reviewed in [72]), further research on the role of YB-1 in human disease pathogenesis may pay off in the long run.

### Conclusion

In conclusion, we confirm that the nucleic acid binding YB-1 is an important cofactor of HIV replication that exerts its effects at two stages in a concentration-dependent manner. The early effect occurs at a stage between reverse transcription and nuclear import. The late effect involves viral RNA stability and virus production.

## Supporting information

S1 Table Primer and adaptor sequences used in this manuscript. (DOCX)

S2 Table miRNA based target sequences used in this manuscript. (DOCX)

S3 Table qPCR primers and probes used in this manuscript. (DOCX)

S4 Table Putative cellular co-factors of HIV-1 integrase. (DOCX)

S5 Table HIV integration site distribution. (DOCX)

S1 Fig Overview of the plasmid constructs. Shown is a schematic representation of the cassettes on (**A**) pGAE_sffv_LoxP_BsdR_miR_WPRE_LoxP, to induce miRNA-based shRNA-mediated knockdown of YB-1, (**B**) the resulting product after delivery of Cre recombinase in stable cell lines expressing the construct shown in A, (**C**) pGAE_sffv_YB-1s_IRES_HygroR_WPRE expressing miR-resistant YB-1 used to backcomplement the YB-1 knockdown cell lines and (**D**) pGAE_sffv_MCS_IRES_HygroR_WPRE used as a control for the backcomplementation.
(PDF)

S2 Fig Binding of YB-1 to different proteins. Interaction of a dilution series of recombinant (A) GST-YB-1 with 80 nM His-integrase (IN) or 2 nM JPO2-MBP or (B) the YB-1 cold shock domain (GST-YB-1 CSD) with 80 nM His-IN, 100 nM His-Transportin SR-2 (TRN), His-LEDGF/p75, His-LEDGF/p52 and His-Menin. Proteins were purified as described in [73] and in the Materials and Methods section.
(TIF)

S3 Fig YB-1 depletion reduces viral particle production and viral RNA levels. Transfection of HeLaP4-derived cell lines with plasmid encoding for (**A, C, D, E, F**) HIV_YFP_ (pHIV_YFP_) or (**B**) HIV_NL4.3_ (pHIV). (**A**) Transfection with pHIV_YFP_ in cell lines expressing YB-1 miR 1 (miRY1, *red*) or miR 2 (miRY2, *dark blue*) compared to control cell lines (WT, *light grey* or cells expressing a control miR, miRctrl, *dark grey*), as measured with p24 ELISA. 2 μM ritonavir (RTV) was added as a positive control. YB-1 depletion was rescued through overexpression of miR resistant YB-1 (miRY1+BC, *violet*). Backomplementation was controlled with an empty vector construct (miRY1+ctrl, *pink*). (**B**) Similar results were obtained upon HIV_NL4.3_ transfection. (**C**) Total viral Gag RNA levels as measured with RT-qPCR in HelaP4 cells depleted for YB-1 (*red*) or miRctrl cells (*dark grey*). (**D**, **E**, **F**) YFP expression levels were measured by flow cytometry upon transfection with (**D**) 30 ng or (**E**, **F**) 60 ng pHIV_YFP_ in 96-well plates. (**D**) This experiment was performed in parallel with the experiment in **Fig 3C** with a lower amount of plasmid. (**E**) Cells were transduced with different quantities of vector encoding for miRY2 (*blue*) and/or by a dilution series of a vector coding for a YB-1 backcomplementation (vector YB-1 BC: miRY2+BC, *green*). The respective YB-1 RNA expression levels are shown in Fig 4D. (**A**-**D**, **F**) Results were compared to results obtained with the miRctrl (*black stars*) or the BC conditions (*violet* or *green stars*). (**E**) Results were compared to results obtained with miRY1 (*dark blue stars*) or miRY1+ctrl (*light blue*) conditions. *p <0.*05*,**p < 0.*01*, ***p < 0.*001*.
(PDF)

S4 Fig YB-1 depletion reduces also gag p55 expression. Transfection with pHIV_YFP_ in HeLaP4-derived cell lines expressing YB-1 miR 1 (miRY1, *red*) or miR 2 (miRY2, *dark blue*) compared to control cell lines (WT, *light grey* or cells expressing a control miR, miRctrl, *dark grey*), followed by Western blotting analysis of these cell lysates using anti-capsid (anti-CA) and anti-integrase (anti-IN) antibodies. Membranes were washed between anti-CA and anti-IN staining, resulting in the observation of CA-containing bands in the anti-IN blot. Β-actin was detected after stripping. Relative gag p55/ β-actin intensities were quantified using the ImageJ intensity analysis. Error bars indicate standard deviations from two independent experiments. Differences were determined using one-way ANOVA, followed by the Bonferroni multiple comparison test. Results were compared to results obtained with the miRctrl (*black stars*) or the BC conditions (*violet stars*). *p <0.*05*,**p < 0.*01*, ***p < 0.*001*. Integrase staining was added as an additional control.
(PDF)

S5 Fig YB-1 levels determine HIV-1 transduction efficiency. (**A, B**) HeLaP4 cells transduced with a dilution series of vector expressing a miRNA-based shRNA against YB-1 (miRY1, *red*, miRY2, *blue*) or miRNA-based shRNA against DSRed (miRctrl, *dark grey*) were transduced with a dilution series of VSV-G pseudotyped HIV_YFP_ and harvested 48–72 hours post transduction. % YFP positive cells (left panel) and the % YFP positive cells x mean fluorescence intensity (MFI) (right panel) as measured by flow cytometry are depicted. Shown is a representative experiment out of 3 independent experiments. (**C, D**) HeLaP4 cells with a stable YB-1 knock-down (miRY2) were transduced and selected with a dilution series of vector expressing miR resistant YB-1 (miRY2+BC, *green*) or control vector (miRY2+ctrl, *light blue*) prior to infection with different amounts (125 ng p24, **C** or 14 ng p24, **D**) of single round VSV-G pseudotyped HIV_YFP_. Cells were harvested 48–72 hours post transduction. % YFP positive cells (left panel) and the % YFP positive cells x mean fluorescence intensity (MFI) (right panel) as measured by flow cytometry are depicted. (**E**) Similar results were obtained upon rescue of the YB-1 levels after mirY1 KD (miRY1+BC, *violet*). A representative experiment out of 2 to 4 experiments is shown. Error bars represent standard deviations of triplicate data points. Statistical differences were determined using one-way ANOVA, followed by the Bonferroni multiple comparison test. Cells were compared to (**A**, **B**, **C**) miRctrl (*black stars*) and miRY2+BC (*green stars*), (**C**-**E**) miRY1 (*red stars*), miRY2 (*dark blue stars*), miRY1+ctrl (*pink stars*) or miRY2+ctrl (*light blue stars*) conditions. *p <0.*05*,**p < 0.*01*, ***p < 0.*001*.
(PDF)

S6 Fig Total number and nuclear membrane localisation of IN-eGFP HIV particles. HeLaP4 YB-1 knock-down (miRY1, *red*, n = 38) or control (miRctrl, *dark grey*, n = 40) cells were infected with fluorescent HIV_IN-eGFP_ and fixed 5 hours post transduction. As a backcomplementationcontrol the miRY1 was floxed out using Cre–recombinase (miRY1+cre, *violet*, n = 35). Green fluorescent particles in the cytoplasm and nucleus were quantified by laser-scanning confocal microscopy. The numbers of PICs per (**A**) cell and at the (**B**) nuclear membrane are shown. Error bars represent standard deviations. No statistical significant differences between miRctrl, miRY1 and miRY1+cre conditions were observed using (**A**) one-way ANOVA or (**B**) Mann-Withney testing. One out of two representative experiments is shown. *p <0.*05*,**p < 0.*01*, ***p < 0.*001*.
(PDF)

S7 Fig MLV transduction efficiency. The stable HeLaP4 YB-1 KD cell lines (miRY2) and respective control cell lines were transduced with a VSV-G pseudotyped MLV-vector encoding for eGFP and results were analyzed using flow cytometry. A representative experiment out of 2 experiments is shown. Error bars represent standard deviations of triplicate data points. Statistical differences were determined using one-way ANOVA, followed by the Bonferroni multiple comparison test. Cells were compared to (**A**, **B**, **C**) miRctrl (*black stars*) and miRY2+BC (*green stars*), (**C**-**E**) miRY1 (*red stars*), miRY2 (*dark blue stars*), miRY1+ctrl (*pink stars*) or miRY2+ctrl (*light blue stars*) conditions. *p <0.*05*,**p < 0.*01*, ***p < 0.*001*.
(PDF)

## References

[1] Barre-Sinoussi F, Ross AL, Delfraissy JF. Past, present and future: 30 years of HIV research. Nat Rev Microbiol. 2013;11(12):877–83. Epub 2013/10/29. doi: 10.1038/nrmicro3132 . 24162027 10.1038/nrmicro3132

[2] Klatzmann D, Champagne E, Chamaret S, Gruest J, Guetard D, Hercend T, et al T-lymphocyte T4 molecule behaves as the receptor for human retrovirus LAV. Nature. 1984;312(5996):767–8. Epub 1984/12/20. . 6083454 10.1038/312767a0

[3] Alkhatib G, Combadiere C, Broder CC, Feng Y, Kennedy PE, Murphy PM, et al CC CKR5: a RANTES, MIP-1alpha, MIP-1beta receptor as a fusion cofactor for macrophage-tropic HIV-1. Science. 1996;272(5270):1955–8. Epub 1996/06/28. . 8658171 10.1126/science.272.5270.1955

[4] Feng Y, Broder CC, Kennedy PE, Berger EA. HIV-1 entry cofactor: functional cDNA cloning of a seven-transmembrane, G protein-coupled receptor. Science. 1996;272(5263):872–7. Epub 1996/05/10. . 8629022 10.1126/science.272.5263.872

[5] Matreyek KA, Engelman A. The requirement for nucleoporin NUP153 during human immunodeficiency virus type 1 infection is determined by the viral capsid. J Virol. 2011;85(15):7818–27. Epub 2011/05/20. doi: 10.1128/JVI.00325-11 ; PubMed Central PMCID: PMC3147902. 21593146 10.1128/JVI.00325-11 PMC3147902

[6] Cherepanov P, Maertens G, Proost P, Devreese B, Van Beeumen J, Engelborghs Y, et al HIV-1 integrase forms stable tetramers and associates with LEDGF/p75 protein in human cells. J Biol Chem. 2003;278(1):372–81. Epub 2002/10/31. doi: 10.1074/jbc.M209278200 . 12407101 10.1074/jbc.M209278200

[7] Neville M, Stutz F, Lee L, Davis LI, Rosbash M. The importin-beta family member Crm1p bridges the interaction between Rev and the nuclear pore complex during nuclear export. Curr Biol. 1997;7(10):767–75. Epub 1997/11/22. . 9368759 10.1016/s0960-9822(06)00335-6

[8] Dalgleish AG, Beverley PC, Clapham PR, Crawford DH, Greaves MF, Weiss RA. The CD4 (T4) antigen is an essential component of the receptor for the AIDS retrovirus. Nature. 1984;312(5996):763–7. Epub 1984/12/20. . 6096719 10.1038/312763a0

[9] U.S. Food and Drug Administration. Drugs@FDA 2015 [cited 2015 March 8]. Available from: http://www.accessdata.fda.gov/.

[10] Christ F, Voet A, Marchand A, Nicolet S, Desimmie BA, Marchand D, et al Rational design of small-molecule inhibitors of the LEDGF/p75-integrase interaction and HIV replication. Nat Chem Biol. 2010;6(6):442–8. Epub 2010/05/18. doi: 10.1038/nchembio.370 . 20473303 10.1038/nchembio.370

[11] Fenwick C, Amad M, Bailey MD, Bethell R, Bos M, Bonneau P, et al Preclinical profile of BI 224436, a novel HIV-1 non-catalytic-site integrase inhibitor. Antimicrobial agents and chemotherapy. 2014;58(6):3233–44. Epub 2014/03/26. doi: 10.1128/AAC.02719-13 ; PubMed Central PMCID: PMC4068430. 24663024 10.1128/AAC.02719-13 PMC4068430

[12] Chen H, Li C, Huang J, Cung T, Seiss K, Beamon J, et al CD4+ T cells from elite controllers resist HIV-1 infection by selective upregulation of p21. J Clin Invest. 2011;121(4):1549–60. Epub 2011/03/16. doi: 10.1172/JCI44539 ; PubMed Central PMCID: PMC3069774. 21403397 10.1172/JCI44539 PMC3069774

[13] De Rijck J, de Kogel C, Demeulemeester J, Vets S, El Ashkar S, Malani N, et al The BET family of proteins targets moloney murine leukemia virus integration near transcription start sites. Cell Rep. 2013;5(4):886–94. Epub 2013/11/05. doi: 10.1016/j.celrep.2013.09.040 ; PubMed Central PMCID: PMC4197836. 24183673 10.1016/j.celrep.2013.09.040 PMC4197836

[14] Sharma A, Larue RC, Plumb MR, Malani N, Male F, Slaughter A, et al BET proteins promote efficient murine leukemia virus integration at transcription start sites. Proc Natl Acad Sci U S A. 2013;110(29):12036–41. Epub 2013/07/03. doi: 10.1073/pnas.1307157110 ; PubMed Central PMCID: PMC3718171. 23818621 10.1073/pnas.1307157110 PMC3718171

[15] Gupta SS, Maetzig T, Maertens GN, Sharif A, Rothe M, Weidner-Glunde M, et al Bromo- and extraterminal domain chromatin regulators serve as cofactors for murine leukemia virus integration. J Virol. 2013;87(23):12721–36. Epub 2013/09/21. doi: 10.1128/JVI.01942-13 ; PubMed Central PMCID: PMC3838128. 24049186 10.1128/JVI.01942-13 PMC3838128

[16] Ciuffi A, Llano M, Poeschla E, Hoffmann C, Leipzig J, Shinn P, et al A role for LEDGF/p75 in targeting HIV DNA integration. Nat Med. 2005;11(12):1287–9. Epub 2005/11/29. doi: 10.1038/nm1329 . 16311605 10.1038/nm1329

[17] Lyabin DN, Eliseeva IA, Ovchinnikov LP. YB-1 protein: functions and regulation. Wiley Interdiscip Rev RNA. 2014;5(1):95–110. Epub 2013/11/13. doi: 10.1002/wrna.1200 . 24217978 10.1002/wrna.1200

[18] Uchiumi T, Fotovati A, Sasaguri T, Shibahara K, Shimada T, Fukuda T, et al YB-1 is important for an early stage embryonic development: neural tube formation and cell proliferation. J Biol Chem. 2006;281(52):40440–9. Epub 2006/11/04. doi: 10.1074/jbc.M605948200 . 17082189 10.1074/jbc.M605948200

[19] Lu ZH, Books JT, Ley TJ. YB-1 is important for late-stage embryonic development, optimal cellular stress responses, and the prevention of premature senescence. Mol Cell Biol. 2005;25(11):4625–37. Epub 2005/05/19. doi: 10.1128/MCB.25.11.4625-4637.2005 ; PubMed Central PMCID: PMC1140647. 15899865 10.1128/MCB.25.11.4625-4637.2005 PMC1140647

[20] Wolffe AP. Structural and functional properties of the evolutionarily ancient Y-box family of nucleic acid binding proteins. Bioessays. 1994;16(4):245–51. Epub 1994/04/01. doi: 10.1002/bies.950160407 . 8031301 10.1002/bies.950160407

[21] Didier DK, Schiffenbauer J, Woulfe SL, Zacheis M, Schwartz BD. Characterization of the cDNA encoding a protein binding to the major histocompatibility complex class II Y box. Proc Natl Acad Sci U S A. 1988;85(19):7322–6. Epub 1988/10/01. ; PubMed Central PMCID: PMC282178. 3174636 10.1073/pnas.85.19.7322 PMC282178

[22] Dolfini D, Mantovani R. YB-1 (YBX1) does not bind to Y/CCAAT boxes in vivo. Oncogene. 2013;32(35):4189–90. Epub 2012/11/20. doi: 10.1038/onc.2012.521 . 23160378 10.1038/onc.2012.521

[23] Pisarev AV, Skabkin MA, Thomas AA, Merrick WC, Ovchinnikov LP, Shatsky IN. Positive and negative effects of the major mammalian messenger ribonucleoprotein p50 on binding of 40 S ribosomal subunits to the initiation codon of beta-globin mRNA. J Biol Chem. 2002;277(18):15445–51. Epub 2002/02/21. doi: 10.1074/jbc.M111954200 . 11854282 10.1074/jbc.M111954200

[24] Minich WB, Ovchinnikov LP. Role of cytoplasmic mRNP proteins in translation. Biochimie. 1992;74(5):477–83. Epub 1992/05/01. . 1637873 10.1016/0300-9084(92)90088-v

[25] Nekrasov MP, Ivshina MP, Chernov KG, Kovrigina EA, Evdokimova VM, Thomas AA, et al The mRNA-binding protein YB-1 (p50) prevents association of the eukaryotic initiation factor eIF4G with mRNA and inhibits protein synthesis at the initiation stage. J Biol Chem. 2003;278(16):13936–43. Epub 2003/02/13. doi: 10.1074/jbc.M209145200 . 12582179 10.1074/jbc.M209145200

[26] Kedersha N, Anderson P. Mammalian stress granules and processing bodies. Methods Enzymol. 2007;431:61–81. Epub 2007/10/10. doi: 10.1016/S0076-6879(07)31005-7 . 17923231 10.1016/S0076-6879(07)31005-7

[27] Bann DV, Beyer AR, Parent LJ. A murine retrovirus co-Opts YB-1, a translational regulator and stress granule-associated protein, to facilitate virus assembly. J Virol. 2014;88(8):4434–50. Epub 2014/02/07. doi: 10.1128/JVI.02607-13 ; PubMed Central PMCID: PMC3993753. 24501406 10.1128/JVI.02607-13 PMC3993753

[28] Kawaguchi A, Matsumoto K, Nagata K. YB-1 functions as a porter to lead influenza virus ribonucleoprotein complexes to microtubules. J Virol. 2012;86(20):11086–95. Epub 2012/08/03. doi: 10.1128/JVI.00453-12 ; PubMed Central PMCID: PMC3457152. 22855482 10.1128/JVI.00453-12 PMC3457152

[29] Kashanchi F, Duvall JF, Dittmer J, Mireskandari A, Reid RL, Gitlin SD, et al Involvement of transcription factor YB-1 in human T-cell lymphotropic virus type I basal gene expression. J Virol. 1994;68(1):561–5. Epub 1994/01/01. ; PubMed Central PMCID: PMC236322. 8254772 10.1128/jvi.68.1.561-565.1994 PMC236322

[30] Kerr D, Chang CF, Chen N, Gallia G, Raj G, Schwartz B, et al Transcription of a human neurotropic virus promoter in glial cells: effect of YB-1 on expression of the JC virus late gene. J Virol. 1994;68(11):7637–43. Epub 1994/11/01. ; PubMed Central PMCID: PMC237216. 7933155 10.1128/jvi.68.11.7637-7643.1994 PMC237216

[31] Sawaya BE, Khalili K, Amini S. Transcription of the human immunodeficiency virus type 1 (HIV-1) promoter in central nervous system cells: effect of YB-1 on expression of the HIV-1 long terminal repeat. J Gen Virol. 1998;79 (Pt 2):239–46. Epub 1998/02/24. doi: 10.1099/0022-1317-79-2-239 . 9472608 10.1099/0022-1317-79-2-239

[32] Ozer J, Faber M, Chalkley R, Sealy L. Isolation and characterization of a cDNA clone for the CCAAT transcription factor EFIA reveals a novel structural motif. J Biol Chem. 1990;265(36):22143–52. Epub 1990/12/25. . 1967130

[33] Ansari SA, Safak M, Gallia GL, Sawaya BE, Amini S, Khalili K. Interaction of YB-1 with human immunodeficiency virus type 1 Tat and TAR RNA modulates viral promoter activity. J Gen Virol. 1999;80 (Pt 10):2629–38. Epub 1999/11/26. doi: 10.1099/0022-1317-80-10-2629 . 10573156 10.1099/0022-1317-80-10-2629

[34] Mu X, Li W, Wang X, Gao G. YB-1 stabilizes HIV-1 genomic RNA and enhances viral production. Protein Cell. 2013;4(8):591–7. Epub 2013/04/17. doi: 10.1007/s13238-013-3011-3 . 23589019 10.1007/s13238-013-3011-3 PMC4875536

[35] Li W, Wang X, Gao G. Expression of YB-1 enhances production of murine leukemia virus vectors by stabilizing genomic viral RNA. Protein Cell. 2012;3(12):943–9. Epub 2012/12/12. doi: 10.1007/s13238-012-2090-x . 23225179 10.1007/s13238-012-2090-x PMC4875382

[36] Paranjape SM, Harris E. Y box-binding protein-1 binds to the dengue virus 3'-untranslated region and mediates antiviral effects. J Biol Chem. 2007;282(42):30497–508. Epub 2007/08/30. doi: 10.1074/jbc.M705755200 . 17726010 10.1074/jbc.M705755200

[37] Chatel-Chaix L, Melancon P, Racine ME, Baril M, Lamarre D. Y-box-binding protein 1 interacts with hepatitis C virus NS3/4A and influences the equilibrium between viral RNA replication and infectious particle production. J Virol. 2011;85(21):11022–37. Epub 2011/08/19. doi: 10.1128/JVI.00719-11 ; PubMed Central PMCID: PMC3194978. 21849455 10.1128/JVI.00719-11 PMC3194978

[38] Li Y, Frederick KM, Haverland NA, Ciborowski P, Belshan M. Investigation of the HIV-1 Matrix interactome during virus replication. Proteomics Clin Appl. 2015 Epub 2015/09/12. doi: 10.1002/prca.201400189 . 26360636 10.1002/prca.201400189 PMC4738181

[39] Kappes J, Wu X. Cell-based method and assay for measuring the infectivity and drug sensitivity of immunodeficiency virus. Google Patents; 2005.

[40] Desimmie BA, Weydert C, Schrijvers R, Vets S, Demeulemeester J, Proost P, et al HIV-1 IN/Pol recruits LEDGF/p75 into viral particles. Retrovirology. 2015;12:16 Epub 2015/03/27. doi: 10.1186/s12977-014-0134-4 ; PubMed Central PMCID: PMC4357141. 25809198 10.1186/s12977-014-0134-4 PMC4357141

[41] Sun D, Melegari M, Sridhar S, Rogler CE, Zhu L. Multi-miRNA hairpin method that improves gene knockdown efficiency and provides linked multi-gene knockdown. BioTechniques. 2006;41(1):59–63. Epub 2006/07/28. doi: 10.2144/000112203 . 16869514 10.2144/000112203

[42] Osorio L, Gijsbers R, Oliveras-Salva M, Michiels A, Debyser Z, Van den Haute C, et al Viral vectors expressing a single microRNA-based short-hairpin RNA result in potent gene silencing in vitro and in vivo. Journal of biotechnology. 2014;169:71–81. Epub 2013/11/21. doi: 10.1016/j.jbiotec.2013.11.004 . 24252659 10.1016/j.jbiotec.2013.11.004

[43] Busso D, Delagoutte-Busso B, Moras D. Construction of a set Gateway-based destination vectors for high-throughput cloning and expression screening in Escherichia coli. Anal Biochem. 2005;343(2):313–21. Epub 2005/07/05. doi: 10.1016/j.ab.2005.05.015 . 15993367 10.1016/j.ab.2005.05.015

[44] Geraerts M, Michiels M, Baekelandt V, Debyser Z, Gijsbers R. Upscaling of lentiviral vector production by tangential flow filtration. The journal of gene medicine. 2005;7(10):1299–310. Epub 2005/05/21. doi: 10.1002/jgm.778 . 15906396 10.1002/jgm.778

[45] De Houwer S, Demeulemeester J, Thys W, Rocha S, Dirix L, Gijsbers R, et al The HIV-1 integrase mutant R263A/K264A is 2-fold defective for TRN-SR2 binding and viral nuclear import. J Biol Chem. 2014;289(36):25351–61. Epub 2014/07/27. doi: 10.1074/jbc.M113.533281 ; PubMed Central PMCID: PMC4155696. 25063804 10.1074/jbc.M113.533281 PMC4155696

[46] Albanese A, Arosio D, Terreni M, Cereseto A. HIV-1 pre-integration complexes selectively target decondensed chromatin in the nuclear periphery. PLoS One. 2008;3(6):e2413 Epub 2008/06/12. doi: 10.1371/journal.pone.0002413 ; PubMed Central PMCID: PMC2398779. 18545681 10.1371/journal.pone.0002413 PMC2398779

[47] Brass AL, Dykxhoorn DM, Benita Y, Yan N, Engelman A, Xavier RJ, et al Identification of host proteins required for HIV infection through a functional genomic screen. Science. 2008;319(5865):921–6. Epub 2008/01/12. doi: 10.1126/science.1152725 . 18187620 10.1126/science.1152725

[48] Schambach A, Mueller D, Galla M, Verstegen MM, Wagemaker G, Loew R, et al Overcoming promoter competition in packaging cells improves production of self-inactivating retroviral vectors. Gene Ther. 2006;13(21):1524–33. Epub 2006/06/10. doi: 10.1038/sj.gt.3302807 . 16763662 10.1038/sj.gt.3302807

[49] Desimmie BA, Schrijvers R, Demeulemeester J, Borrenberghs D, Weydert C, Thys W, et al LEDGINs inhibit late stage HIV-1 replication by modulating integrase multimerization in the virions. Retrovirology. 2013;10:57 Epub 2013/06/01. doi: 10.1186/1742-4690-10-57 ; PubMed Central PMCID: PMC3671127. 23721378 10.1186/1742-4690-10-57 PMC3671127

[50] Schrijvers R, De Rijck J, Demeulemeester J, Adachi N, Vets S, Ronen K, et al LEDGF/p75-independent HIV-1 replication demonstrates a role for HRP-2 and remains sensitive to inhibition by LEDGINs. PLoS Pathog. 2012;8(3):e1002558 Epub 2012/03/08. doi: 10.1371/journal.ppat.1002558 PPATHOGENS-D-11-01640 [pii]. . 22396646 10.1371/journal.ppat.1002558 PMC3291655

[51] Mast J, Nanbru C, van den Berg T, Meulemans G. Ultrastructural changes of the tracheal epithelium after vaccination of day-old chickens with the La Sota strain of Newcastle disease virus. Vet Pathol. 2005;42(5):559–65. Epub 2005/09/08. doi: 10.1354/vp.42-5-559 . 16145202 10.1354/vp.42-5-559

[52] Cherepanov P, Pluymers W, Claeys A, Proost P, De Clercq E, Debyser Z. High-level expression of active HIV-1 integrase from a synthetic gene in human cells. FASEB J. 2000;14(10):1389–99. Epub 2000/07/06. . 10877832 10.1096/fj.14.10.1389

[53] Konig R, Zhou Y, Elleder D, Diamond TL, Bonamy GM, Irelan JT, et al Global analysis of host-pathogen interactions that regulate early-stage HIV-1 replication. Cell. 2008;135(1):49–60. Epub 2008/10/16. doi: 10.1016/j.cell.2008.07.032 ; PubMed Central PMCID: PMC2628946. 18854154 10.1016/j.cell.2008.07.032 PMC2628946

[54] Demeulemeester J, Tintori C, Botta M, Debyser Z, Christ F. Development of an AlphaScreen-based HIV-1 integrase dimerization assay for discovery of novel allosteric inhibitors. J Biomol Screen. 2012;17(5):618–28. Epub 2012/02/18. doi: 10.1177/1087057111436343 . 22337657 10.1177/1087057111436343

[55] Christ F, Thys W, De Rijck J, Gijsbers R, Albanese A, Arosio D, et al Transportin-SR2 imports HIV into the nucleus. Curr Biol. 2008;18(16):1192–202. Epub 2008/08/30. doi: 10.1016/j.cub.2008.07.079 . 18722123 10.1016/j.cub.2008.07.079

[56] Thys W, De Houwer S, Demeulemeester J, Taltynov O, Vancraenenbroeck R, Gerard M, et al Interplay between HIV entry and transportin-SR2 dependency. Retrovirology. 2011;8:7 Epub 2011/02/01. doi: 10.1186/1742-4690-8-7 ; PubMed Central PMCID: PMC3041740. 21276267 10.1186/1742-4690-8-7 PMC3041740

[57] Hazuda DJ, Felock P, Witmer M, Wolfe A, Stillmock K, Grobler JA, et al Inhibitors of strand transfer that prevent integration and inhibit HIV-1 replication in cells. Science. 2000;287(5453):646–50. Epub 2000/01/29. . 10649997 10.1126/science.287.5453.646

[58] Holm PS, Bergmann S, Jurchott K, Lage H, Brand K, Ladhoff A, et al YB-1 relocates to the nucleus in adenovirus-infected cells and facilitates viral replication by inducing E2 gene expression through the E2 late promoter. J Biol Chem. 2002;277(12):10427–34. Epub 2002/01/15. doi: 10.1074/jbc.M106955200 . 11788582 10.1074/jbc.M106955200

[59] Jager S, Cimermancic P, Gulbahce N, Johnson JR, McGovern KE, Clarke SC, et al Global landscape of HIV-human protein complexes. Nature. 2012;481(7381):365–70. Epub 2011/12/23. doi: 10.1038/nature10719 ; PubMed Central PMCID: PMC3310911. 22190034 10.1038/nature10719 PMC3310911

[60] Ocwieja KE, Brady TL, Ronen K, Huegel A, Roth SL, Schaller T, et al HIV integration targeting: a pathway involving Transportin-3 and the nuclear pore protein RanBP2. PLoS Pathog. 2011;7(3):e1001313 Epub 2011/03/23. doi: 10.1371/journal.ppat.1001313 ; Central PMCID: PMC3053352. 21423673 10.1371/journal.ppat.1001313 PMC3053352

[61] Roe T, Reynolds TC, Yu G, Brown PO. Integration of murine leukemia virus DNA depends on mitosis. EMBO J. 1993;12(5):2099–108. Epub 1993/05/01. ; PubMed Central PMCID: PMC413431. 8491198 10.1002/j.1460-2075.1993.tb05858.x PMC413431

[62] Lewis PF, Emerman M. Passage through mitosis is required for oncoretroviruses but not for the human immunodeficiency virus. J Virol. 1994;68(1):510–6. Epub 1994/01/01. ; PubMed Central PMCID: PMC236313. 8254763 10.1128/jvi.68.1.510-516.1994 PMC236313

[63] Suzuki Y, Craigie R. The road to chromatin—nuclear entry of retroviruses. Nat Rev Microbiol. 2007;5(3):187–96. Epub 2007/02/17. doi: 10.1038/nrmicro1579 . 17304248 10.1038/nrmicro1579

[64] Ambrose Z, Aiken C. HIV-1 uncoating: connection to nuclear entry and regulation by host proteins. Virology. 2014;454–455:371–9. Epub 2014/02/25. doi: 10.1016/j.virol.2014.08.018 PMID: 25243334; PubMed Central PMCID: PMC3988234. 24559861 10.1016/j.virol.2014.02.004 PMC3988234

[65] Krishnamoorthy G, Roques B, Darlix JL, Mely Y. DNA condensation by the nucleocapsid protein of HIV-1: a mechanism ensuring DNA protection. Nucleic Acids Res. 2003;31(18):5425–32. Epub 2003/09/05. doi: 10.1093/nar/gkg738 ; PubMed Central PMCID: PMC203321. 12954779 10.1093/nar/gkg738 PMC203321

[66] Lyonnais S, Gorelick RJ, Heniche-Boukhalfa F, Bouaziz S, Parissi V, Mouscadet JF, et al A protein ballet around the viral genome orchestrated by HIV-1 reverse transcriptase leads to an architectural switch: from nucleocapsid-condensed RNA to Vpr-bridged DNA. Virus Res. 2013;171(2):287–303. Epub 2012/09/29. doi: 10.1016/j.virusres.2012.09.008 ; PubMed Central PMCID: PMC3552025. 23017337 10.1016/j.virusres.2012.09.008 PMC3552025

[67] Hamid FB, Kim J, Shin CG. Cellular and viral determinants of retroviral nuclear entry. Can J Microbiol. 2016;62(1):1–15. Epub 2015/11/11. doi: 10.1139/cjm-2015-0350 . 26553381 10.1139/cjm-2015-0350

[68] Weydert C, De Rijck J, Christ F, Debyser Z. Targeting virus-host interactions of HIV replication. Curr Top Med Chem. 2015 Epub 2015/09/02. . 26324041 10.2174/1568026615666150901115106

[69] Oda Y, Sakamoto A, Shinohara N, Ohga T, Uchiumi T, Kohno K, et al Nuclear expression of YB-1 protein correlates with P-glycoprotein expression in human osteosarcoma. Clin Cancer Res. 1998;4(9):2273–7. Epub 1998/09/25. . 9748149

[70] Shibao K, Takano H, Nakayama Y, Okazaki K, Nagata N, Izumi H, et al Enhanced coexpression of YB-1 and DNA topoisomerase II alpha genes in human colorectal carcinomas. Int J Cancer. 1999;83(6):732–7. Epub 1999/12/22. . 10597187 10.1002/(sici)1097-0215(19991210)83:6<732::aid-ijc6>3.0.co;2-#

[71] Kamura T, Yahata H, Amada S, Ogawa S, Sonoda T, Kobayashi H, et al Is nuclear expression of Y box-binding protein-1 a new prognostic factor in ovarian serous adenocarcinoma? Cancer. 1999;85(11):2450–4. Epub 1999/06/05. . 10357417 10.1002/(sici)1097-0142(19990601)85:11<2450::aid-cncr21>3.0.co;2-u

[72] Kosnopfel C, Sinnberg T, Schittek B. Y-box binding protein 1—a prognostic marker and target in tumour therapy. European Journal of Cell Biology. 2014;93(1–2):61–70. Epub 2014/01/28. doi: 10.1016/j.ejcb.2013.11.007 . 24461929 10.1016/j.ejcb.2013.11.007

[73] Tesina P, Čermáková K, Hořejší M, Procházková K, Fábry M, Sharma S, et al Multiple cellular proteins interact with LEDGF/p75 through a conserved unstructured consensus motif. Nat Commun. 2015;6:7968 doi: 10.1038/ncomms8968 26245978 10.1038/ncomms8968

