# Correction: Y-box-binding protein 1 supports the early and late steps of HIV replication

**DOI:** 10.1371/journal.pone.0241893

**Published:** 2020-10-29

**Authors:** Caroline Weydert, Bart van Heertum, Lieve Dirix, Stéphanie De Houwer, Flore De Wit, Jan Mast, Steven J. Husson, Katrien Busschots, Renate König, Rik Gijsbers, Jan De Rijck, Zeger Debyser

Panel B of Fig 4 is incorrect. The authors have provided a corrected version of Fig 4 here.

**Fig 4:**
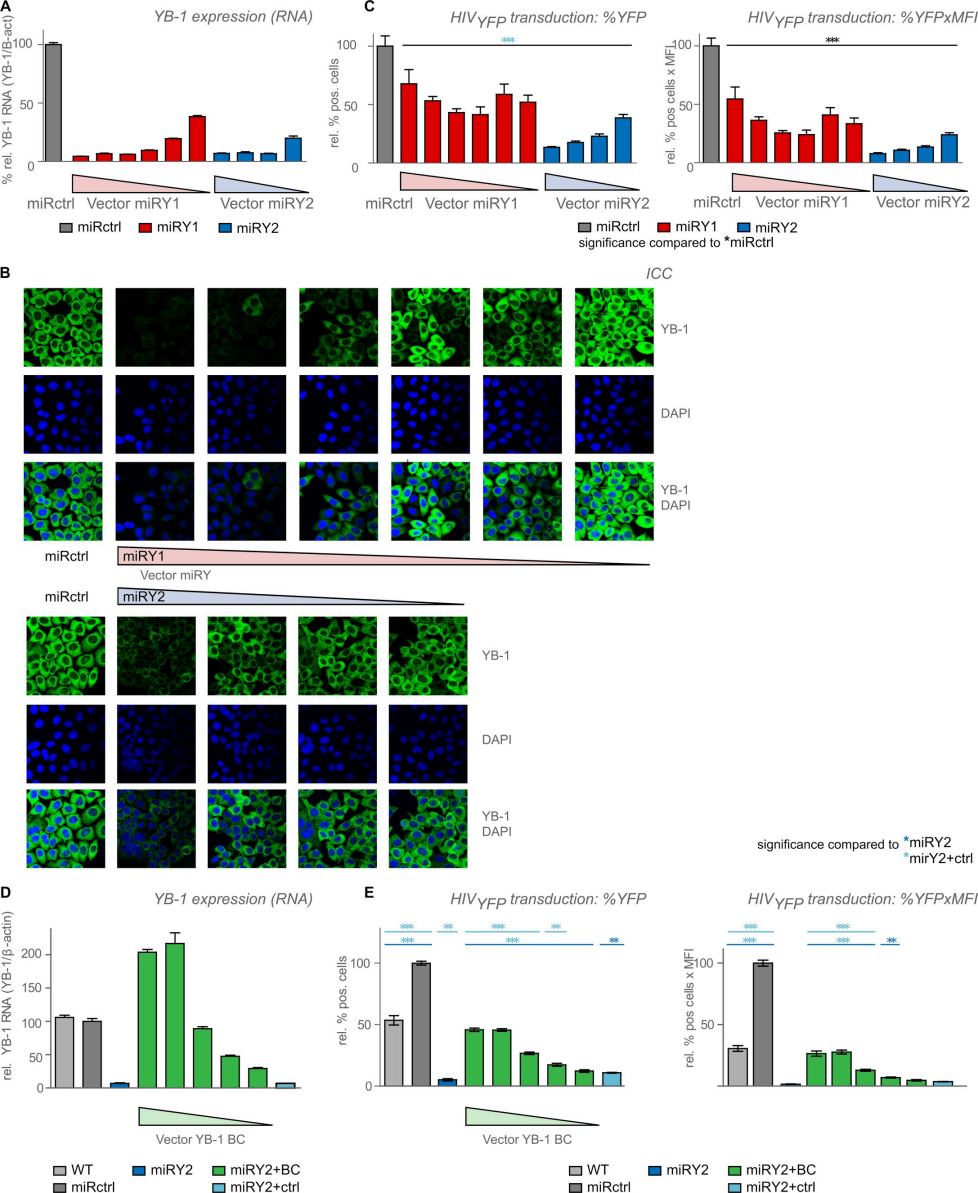
YB-1 levels affect the early steps of HIV replication. HeLaP4 cells were transduced with a dilution series of vector expressing a miRNA-based shRNA against YB-1 (miRY1, *red*, miRY2, *blue*) or against DSRed (miRctrl, *dark grey*). YB-1 expression levels were measured with (**A**) RT-qPCR and with (**B**) immunocytochemistry (ICC). YB-1 was detected with a specific antibody (*green*). DNA was stained using DAPI. (**C**) The stable cell lines were infected with VSV-G pseudotyped HIV_YFP_ and harvested 48–72 hours post infection. % YFP positive cells (*upper panel*) and the % YFP positive x mean fluorescence intensity (MFI) (*lower panel*) were measured using flow cytometry. Shown is a representative experiment out of 3 independent experiments. Standard deviations for triplicates within one experiment are shown. The various YB-1-depleted cell lines were compared to the miRctrl (*black stars*) condition using one way ANOVA followed by the Bonferroni multiple comparison test. (**D**) HeLaP4 cells depleted for YB-1 were transduced with different concentrations of vector expressing miRNA-resistant YB-1 (miRY2+BC, *green*) or control vector (miRY2+ctrl, *light blue*) and RNA expression levels were determined with RT-qPCR. (**E**) Experiment performed as in panel C. VSV-G pseudotyped HIV_YFP_ infection efficiency was partially restored upon YB-1 backcomplementation, as measured by flow cytometry. Shown is a representative experiment out of 3 independent experiments. Standard deviations of triplicate data points are shown. Differences were determined using one-way ANOVA, followed by the Bonferroni multiple comparison test. The miRctrl and the BC dilution series were compared to the miRY2 (*dark blue stars*) and miRY2+ctrl (*light blue stars*) conditions. *p <0.*05*,**p < 0.*01*, ***p < 0.*001*.
