# Supplementary material for: Y-box-binding protein 1 supports the early and late steps of HIV replication
Source: PLoS One. 2018 Jul 11;13(7):e0200080. doi: 10.1371/journal.pone.0200080 (PMC6040738; doi:10.1371/journal.pone.0200080)
Supplement: S1 Table — (DOCX) [file pone.0200080.s001.docx]

**Table S1: Primer and adaptor sequences used in this manuscript.**

|  | forward | reverse |
| --- | --- | --- |
| miR30stemshuttle adaptor | AGCTCTCGAGATCCAAGAAGGTATATTGCTGTTGACAGTGAGCGGAGACGAAGCTTCGTCTCTGCCTACTGCCTCGGACTTCAAGGGCTACGATG | TCGACATCGTAGCCCTTGAAGTCCGAGGCAGTAGGCAGAGACGAAGCTTCGTCTCCGCTCACTGTCAACAGCAATATACCTTCTTGGATCTCGAG |
| BsdR | AAAAAACCGGTATGGCCAAGCCTTTGTCTCAAGA | AAAAAACTCGAGTTAGCCCTCCCACACATAACCAG |
| miRNA-adaptor | GAGCG-miRsense(s)-TAGTGAAGCCACAGATGTA –miRantisense(as)-T | AGGCA-miRantisense (reverse complement)-TACATCTGTGGCTTCACT-miRsense (reverse complement)-C |
| *LoxP*(left) adaptor | GATCATAACTTCGTATAGCATACATTATACGAAGTTAT A | CCGGTATAACTTCGTATAATGTATGCTATACGAAGTTAT |
| *LoxP*(right) adaptor | TCAG ATAACTTCGTATA GCATACAT TATACGAAGTTAT | TGAATAACTTCGTATAATGTATGCTATACGAAGTTATC |
| YBX1s primers | AAAACTCGAGATGAGCAGCGAGGCCGAGAC | TTTTAGATCTTTACTCAGCCCCGCC |
