# Supplementary material for: Y-box-binding protein 1 supports the early and late steps of HIV replication
Source: PLoS One. 2018 Jul 11;13(7):e0200080. doi: 10.1371/journal.pone.0200080 (PMC6040738; doi:10.1371/journal.pone.0200080)
Supplement: S2 Table — (DOCX) [file pone.0200080.s002.docx]

**Table S2: MiRNA based target sequences used in this manuscript.**

|  | sense | antisense |
| --- | --- | --- |
| miRctrl | TGTTCAATGTAAGGAACGGATA | TATCCGTTCCTTACATTGAACC |
| miRY1 | GCCAGTTCAAGGCAGTAAATAT | ATATTTACTGCCTTGAACTGGA |
| miRY2 | AAGCAGACCGTAACCATTATAG | CTATAATGGTTACGGTCTGCTG |
| miRLedgf | CAGACAGCATGAGGAAGCGAAT | ATTCGCTTCCTCATGCTGTCTT |
