# Supplementary material for: Y-box-binding protein 1 supports the early and late steps of HIV replication
Source: PLoS One. 2018 Jul 11;13(7):e0200080. doi: 10.1371/journal.pone.0200080 (PMC6040738; doi:10.1371/journal.pone.0200080)
Supplement: S3 Table — (DOCX) [file pone.0200080.s003.docx]

**Table S3: qPCR primers and probes used in this manuscript**

|  | sense | antisense | probe |
| --- | --- | --- | --- |
| YB-1 | CTCGCCAAAGACAGCCTAGA | AATTCTCAGCTGGTGGAT CG | (FAM)-GGAGATGAGACCCAAGGTCA-(TAMRA) |
| Gag (reverse transcripts, integrated copies HIV_IIIB_ and HIV_YFP_) | ATCAAGCAGCCATGCAAATGTT | CTGAAGGGTACTAGTAGTTCCTGCTATGTC | (FAM)-ACCATCAATGAGGAAGCTGCAGAATGGGA-(TAMRA) |
| 2-LTR circles | GTGCCCGTCTGTTGTGTGACT | CTTGTCTTCTTTGGGAGAGAATTAGC | (FAM)-TCCACACTGACTAAAAGGGTCTGAGGGATCTCT-(TAMRA) |
| Wpre  (LV integrated copies) | CCGTTGTCAGGCAACGTG | AGCTGACAGGTGGTGGCAAT | FAM-TGCTGACGCAACCCCcATcGGT-Tamra |
| Luc  (Luc integrated copies) | GAAGAGATACGCCCTGGTTCC | TGTGATTTGTATTCAGCCCATATCG | FAM-TTCATAGCTTCTGCCAACCGAACGGACA-TAMRA |
| B-actin cDNA | CACTGAGCGAGGCTACAGCTT | TTGATGTCGCGCACGATTT | 5'-HEX-ACCACCACGGCCGAGCGG-TAMRA-3' |
