# Supplementary material for: Y-box-binding protein 1 supports the early and late steps of HIV replication
Source: PLoS One. 2018 Jul 11;13(7):e0200080. doi: 10.1371/journal.pone.0200080 (PMC6040738; doi:10.1371/journal.pone.0200080)
Supplement: S4 Table — (DOCX) [file pone.0200080.s004.docx]

**Table S4A: Putative cellular co-factors of HIV‑1 integrase.**

| HIV co-factors | | | | HIV restriction factors | | | |
| --- | --- | --- | --- | --- | --- | --- | --- |
| ID^1^ | **MP**  **Score^2^** | **Loc^3^** | **siRNA^4^** | **ID^1^** | **MP score^2^** | **Loc^3^** | **siRNA^4^** |
| *RBM10* | 146-160 | N + C | 0.45 | ***HSPA1A*** | 160 | C | 1.7 |
| *DHX9* | 142 | N | 0.50 | ***PIAS1*** | 62 | N | 2.3 |
| *PPM1B* | 110 | C | 0.55 | ***BRD4*** | ND |  | 3 |
| *SNRPD3* | 106 | C | 0.60 | ***CDCA7L (JPO2)*** | ND |  | 2 |
| *YB-1* (or *YBX1*) | 86 | C | 0.50 |  |  |  |  |
| *KPNB1* | 80 | N | 0.40 |  |  |  |  |
| *SEC61B* | 73 | N | 0.60 |  |  |  |  |
| *XRCC5* | 71 | C | 0.50 |  |  |  |  |
| *SERBP1* | 71 | C | 0.70 |  |  |  |  |
| *RPN2* | 66 | N | 0.60 |  |  |  |  |
| *HNRNPK* | 41-90 | N + C | 0.30 |  |  |  |  |
| *TAB3* | 57 | C | 0.20 |  |  |  |  |
| *HNRNPU* | 23-33 | N + C | 0.30 |  |  |  |  |
| Control: *PSIP1* (LEDGF/p75) | 172-205 | N | 1.0 |  |  |  |  |
| *PogZ* | ND |  | 0.4 |  |  |  |  |

^1^ Gene name.of the identified proteins ^2^ Mass spectrometry (MP) analysis score of co‑IP specified by MOWSE algorithm. ^3^ Cellular localization of the protein. N: Nuclear, C: Cytoplasmatic ^4^ Residual HIV infection (WT infection is 1.00) after siRNA-mediated depletion of each hit as seen in genome wide RNAi screen of König *et al.(*[*56*](#_ENREF_56)*)*≥ 2 siRNAs reducing HIV infection > 30% were required for inclusion in the list. ND: not determined

**Table S4B: Initial co-factor screen**

| Initial co-factor screen | | | |
| --- | --- | --- | --- |
|  | **YB-1** | **DHX9** | **RBM10** |
| Co-IP with IN^1^ | MLV; HIV‑1 | HIV‑1 | HIV‑1 |
| Co-IP with co-factor^2^ | HIV‑1 | HIV‑1; RSV | HIV‑1; RSV |
| siRNA^3^ | 3 | 3 |  |
| shRNA^3^ | 5 | 5 | 5 |
| Toxicity^4^ | - | +/- | +/- |
| KD^5^ | + | + | + |
| Eff. On HIV-fLuc^6^ | -50% | - | - |
| Eff. On RV-fLuc^6^ | - | 30% | +/- |

^1^ Co‑IP of co-factor with different lenti and retroviral integrases. ^2^ Co‑IP of lentiviral and ɣ-retroviral integrases with co-factor. ^3^ Number of RNAi target sequences tested. ^4^ Toxicity after knockdown of co-factor. ^5^ Knockdown of co-factor analyzed by Western blotting analysis. ^6^ Effect on single round replication of lentiviral or γ-retroviral vectors upon co-factor knockdown.
