# Supplementary material for: Y-box-binding protein 1 supports the early and late steps of HIV replication
Source: PLoS One. 2018 Jul 11;13(7):e0200080. doi: 10.1371/journal.pone.0200080 (PMC6040738; doi:10.1371/journal.pone.0200080)
Supplement: S5 Table — (DOCX) [file pone.0200080.s005.docx]

**Table S5: HIV integration site distribution.**

| Cell line | Vector dilution | Type | Total Sites | % in Refseq gene | % near CpG | % in oncogene |
| --- | --- | --- | --- | --- | --- | --- |
| miRctrl | 1/5 | insertion | 1017 | 75.32 ***\| | 10.13 ***\| | 12 ***\| |
| miRY1 | 1/1 | insertion | 1804 | 76.33 ***\| | 8.15 ***\| | 13.03 ***\| |
| miRctrl + cre | 1/5 | insertion | 1050 | 75.33 ***\| | 8.57 ***\| | 14.48 ***\| |
| miRY1 + cre | 1/5 | insertion | 1266 | 76.07 ***\| | 7.66 **\| | 13.82 ***\| |

HIV-based vector integration sites (LV_eGFP_t2A_fLUC) obtained from HeLaP4 cells and their genomic distribution. The total number of sites, the % of sites in reference genes, in a 4 kilo base window around CpG-islands and in oncogenes are depicted. *** show significant departures (pairwise Fishers test) from matched random controls (MRC) and from the control dataset (*miRctrl*). The significant values/asterisks are separated by '|' in the results table. *****p*< 0.001*. Vector dilutions were matched for similar integrated copy number. All distributions were different from MRC, but not from the control dataset.
