# Supplementary material for: Y-box-binding protein 1 supports the early and late steps of HIV replication
Source: PLoS One. 2018 Jul 11;13(7):e0200080. doi: 10.1371/journal.pone.0200080 (PMC6040738; doi:10.1371/journal.pone.0200080)
Supplement: S1 Fig — Shown is a schematic representation of the cassettes on (A) pGAE_sffv_LoxP_BsdR_miR_WPRE_LoxP, to induce miRNA-based shRNA-mediated knockdown of YB-1, (B) the resulting product after delivery of Cre recombinase in stable cell lines expressing the construct shown in A, (C) pGAE_sffv_YB-1s_IRES_HygroR_WPRE expressing miR-resistant YB-1 used to backcomplement the YB-1 knockdown cell lines and (D) pGAE_sffv_MCS_IRES_HygroR_WPRE used as a control for the backcomplementation. (PDF) [file pone.0200080.s006.pdf]

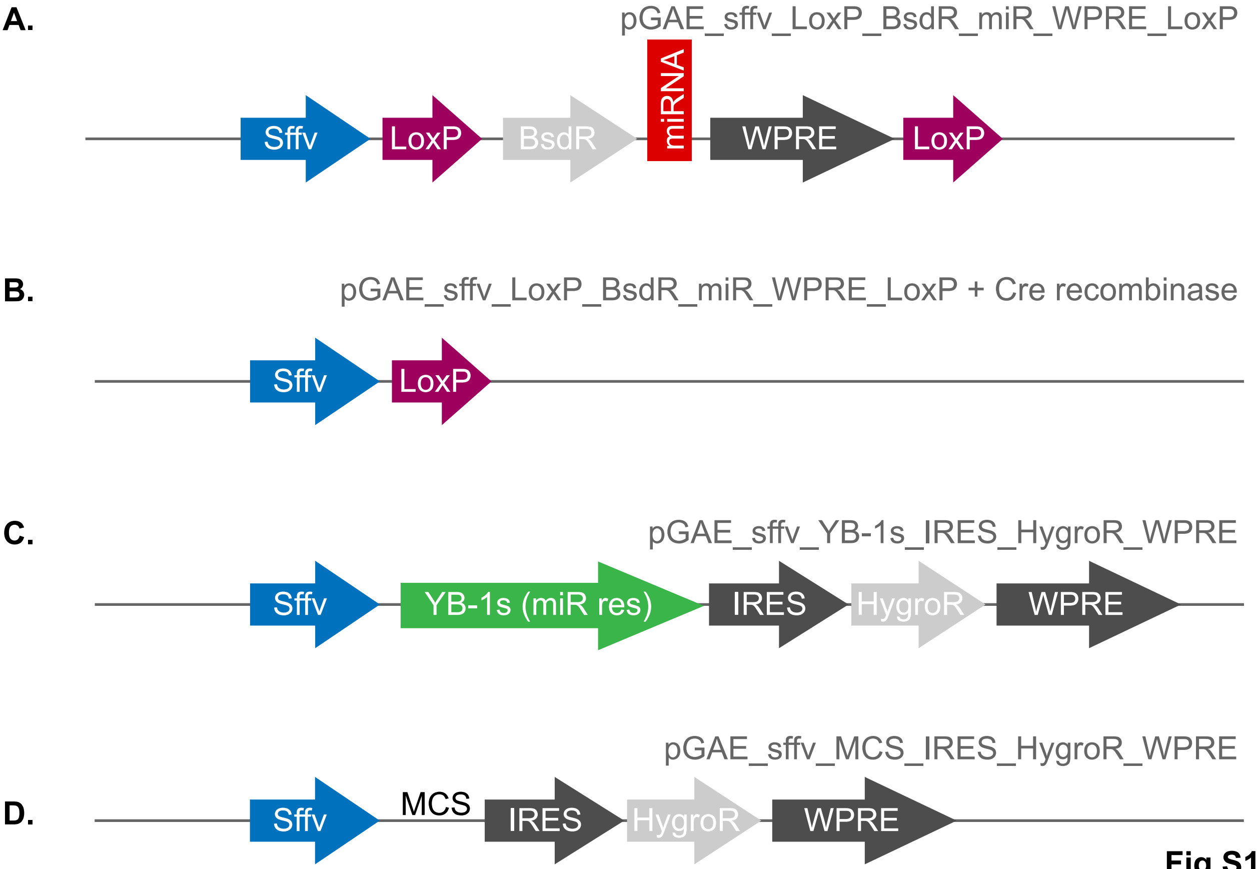
A.
pGAE_sffv_LoxP_BsdR_miR_WPRE_LoxP
Sffv
LoxP
BsdR
miRNA
WPRE
LoxP
B.
pGAE_sffv_LoxP_BsdR_miR_WPRE_LoxP + Cre recombinase
Sffv
LoxP
C.
pGAE_sffv_YB-1s_IRES_HygroR_WPRE
Sffv
YB-1s (miR res)
IRES
HygroR
WPRE
D.
pGAE_sffv_MCS_IRES_HygroR_WPRE
Sffv
MCS
IRES
HygroR
WPRE

Fig S1
