# Supplementary figures and images for: Y-box-binding protein 1 supports the early and late steps of HIV replication
Source: PLoS One. 2018 Jul 11;13(7):e0200080. doi: 10.1371/journal.pone.0200080 (PMC6040738; doi:10.1371/journal.pone.0200080)

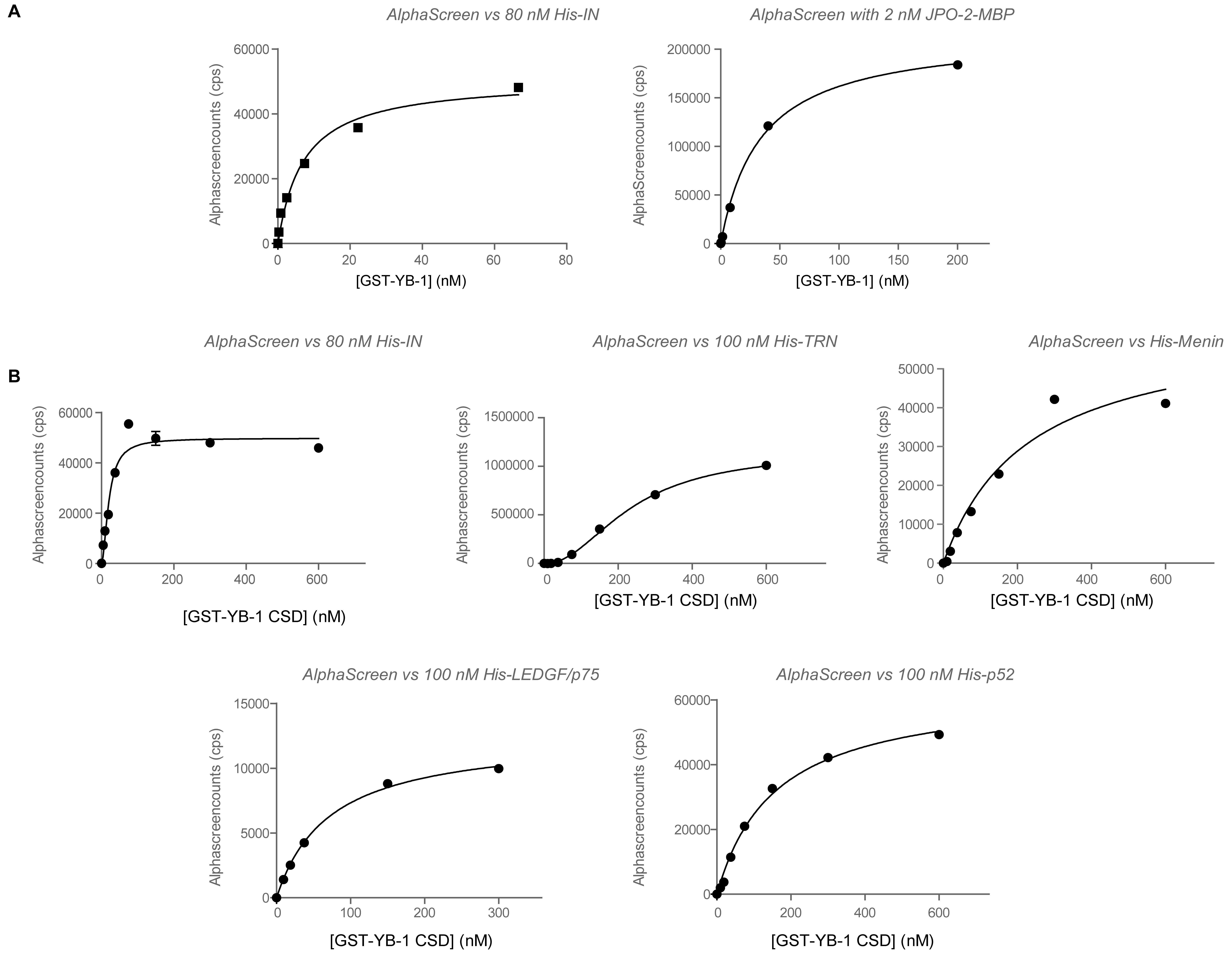

Supplement: S2 Fig — Interaction of a dilution series of recombinant (A) GST-YB-1 with 80 nM His-integrase (IN) or 2 nM JPO2-MBP or (B) the YB-1 cold shock domain (GST-YB-1 CSD) with 80 nM His-IN, 100 nM His-Transportin SR-2 (TRN), His-LEDGF/p75, His-LEDGF/p52 and His-Menin. Proteins were purified as described in [73] and in the Materials and Methods section. (TIF) [file pone.0200080.s007.tif]

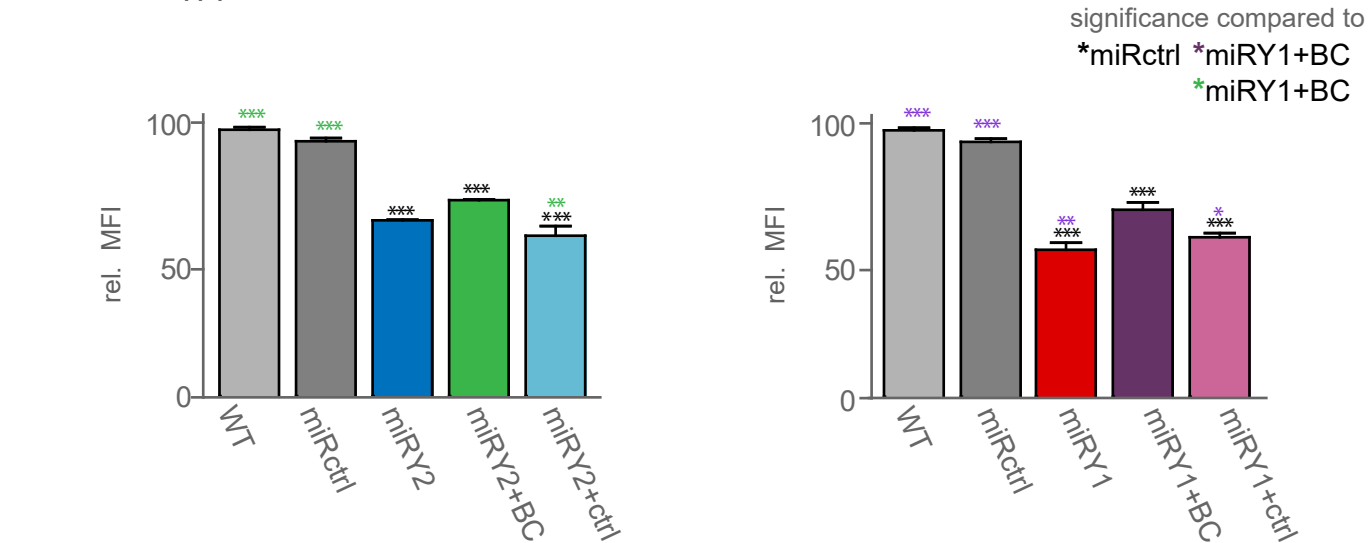

Supplement: S3 Fig — Transfection of HeLaP4-derived cell lines with plasmid encoding for (A, C, D, E, F) HIVYFP (pHIVYFP) or (B) HIVNL4.3 (pHIV). (A) Transfection with pHIVYFP in cell lines expressing YB-1 miR 1 (miRY1, red) or miR 2 (miRY2, dark blue) compared to control cell lines (WT, light grey or cells expressing a control miR, miRctrl, dark grey), as measured with p24 ELISA. 2 μM ritonavir (RTV) was added as a positive control. YB-1 depletion was rescued through overexpression of miR resistant YB-1 (miRY1+BC, violet). Backomplementation was controlled with an empty vector construct (miRY1+ctrl, pink). (B) Similar results were obtained upon HIVNL4.3 transfection. (C) Total viral Gag RNA levels as measured with RT-qPCR in HelaP4 cells depleted for YB-1 (red) or miRctrl cells (dark grey). (D, E, F) YFP expression levels were measured by flow cytometry upon transfection with (D) 30 ng or (E, F) 60 ng pHIVYFP in 96-well plates. (D) This experiment was performed in parallel with the experiment in Fig 3C with a lower amount of plasmid. (E) Cells were transduced with different quantities of vector encoding for miRY2 (blue) and/or by a dilution series of a vector coding for a YB-1 backcomplementation (vector YB-1 BC: miRY2+BC, green). The respective YB-1 RNA expression levels are shown in Fig 4D. (A-D, F) Results were compared to results obtained with the miRctrl (black stars) or the BC conditions (violet or green stars). (E) Results were compared to results obtained with miRY1 (dark blue stars) or miRY1+ctrl (light blue) conditions. *p <0.05,**p < 0.01, ***p < 0.001. (PDF) [file pone.0200080.s008.pdf]

*pHIV<sub>YFP</sub> transfection: gag p55 expression*

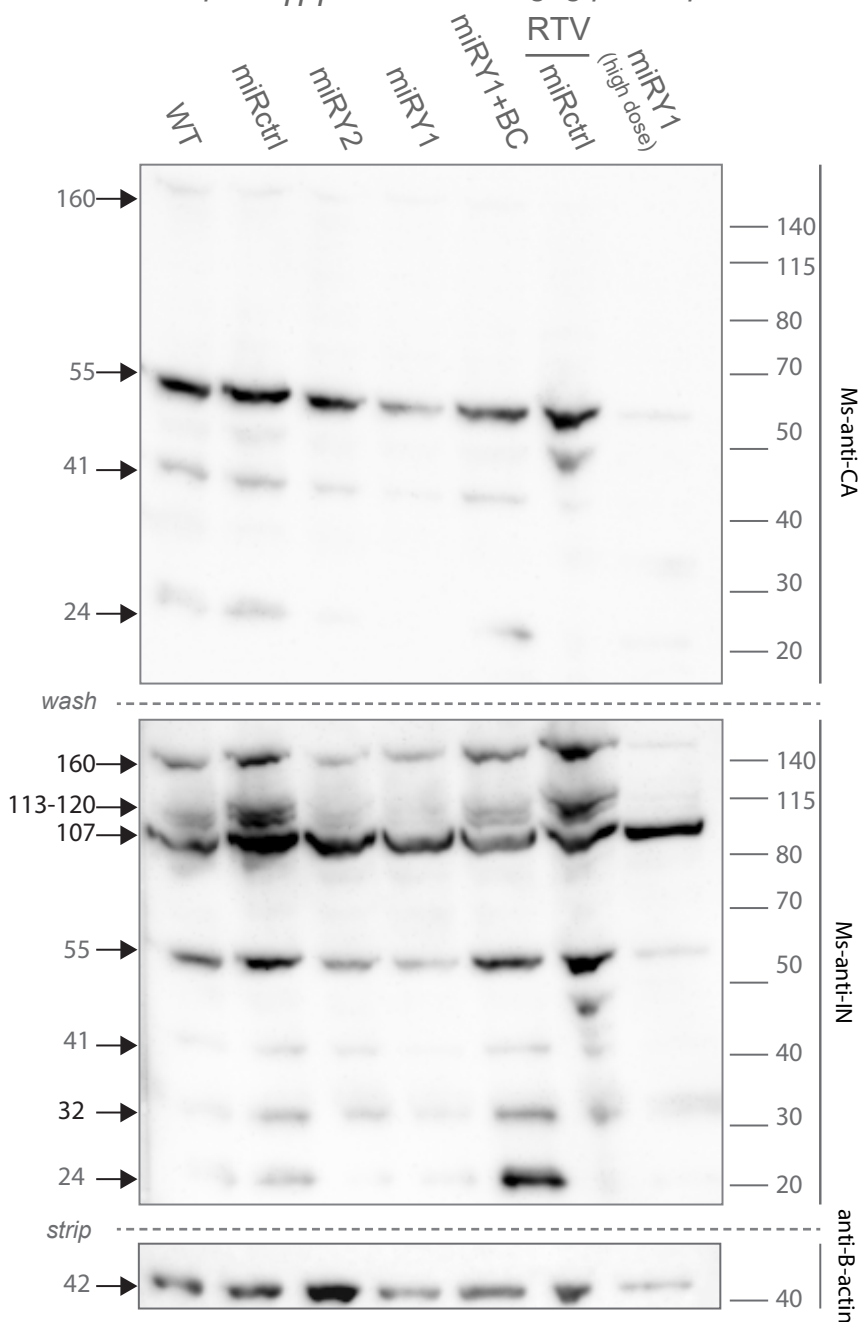

kDa

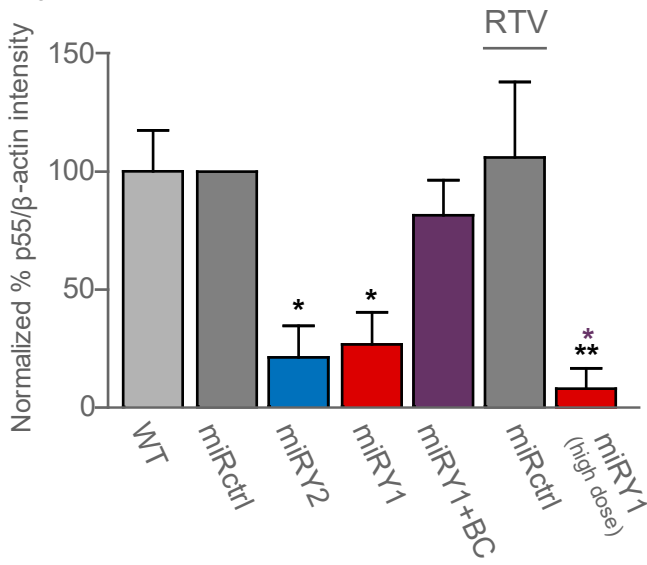

significance compared to  
\*miRctrl \*miRY1+BC  
\*\*

Supplement: S4 Fig — Transfection with pHIVYFP in HeLaP4-derived cell lines expressing YB-1 miR 1 (miRY1, red) or miR 2 (miRY2, dark blue) compared to control cell lines (WT, light grey or cells expressing a control miR, miRctrl, dark grey), followed by Western blotting analysis of these cell lysates using anti-capsid (anti-CA) and anti-integrase (anti-IN) antibodies. Membranes were washed between anti-CA and anti-IN staining, resulting in the observation of CA-containing bands in the anti-IN blot. Β-actin was detected after stripping. Relative gag p55/ β-actin intensities were quantified using the ImageJ intensity analysis. Error bars indicate standard deviations from two independent experiments. Differences were determined using one-way ANOVA, followed by the Bonferroni multiple comparison test. Results were compared to results obtained with the miRctrl (black stars) or the BC conditions (violet stars). *p <0.05,**p < 0.01, ***p < 0.001. Integrase staining was added as an additional control. (PDF) [file pone.0200080.s009.pdf]

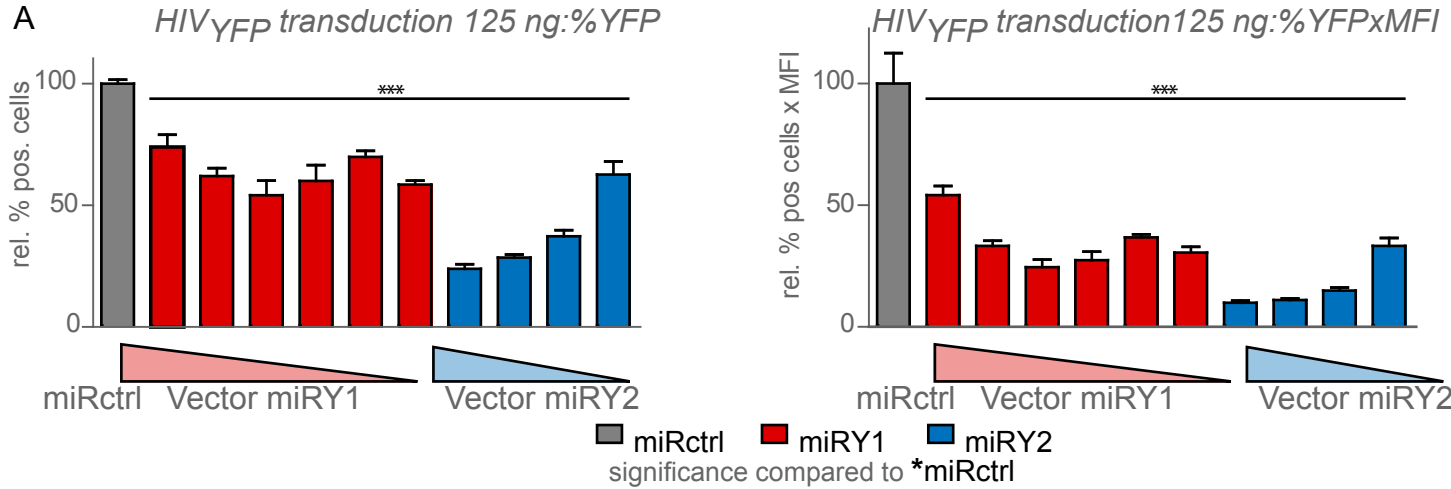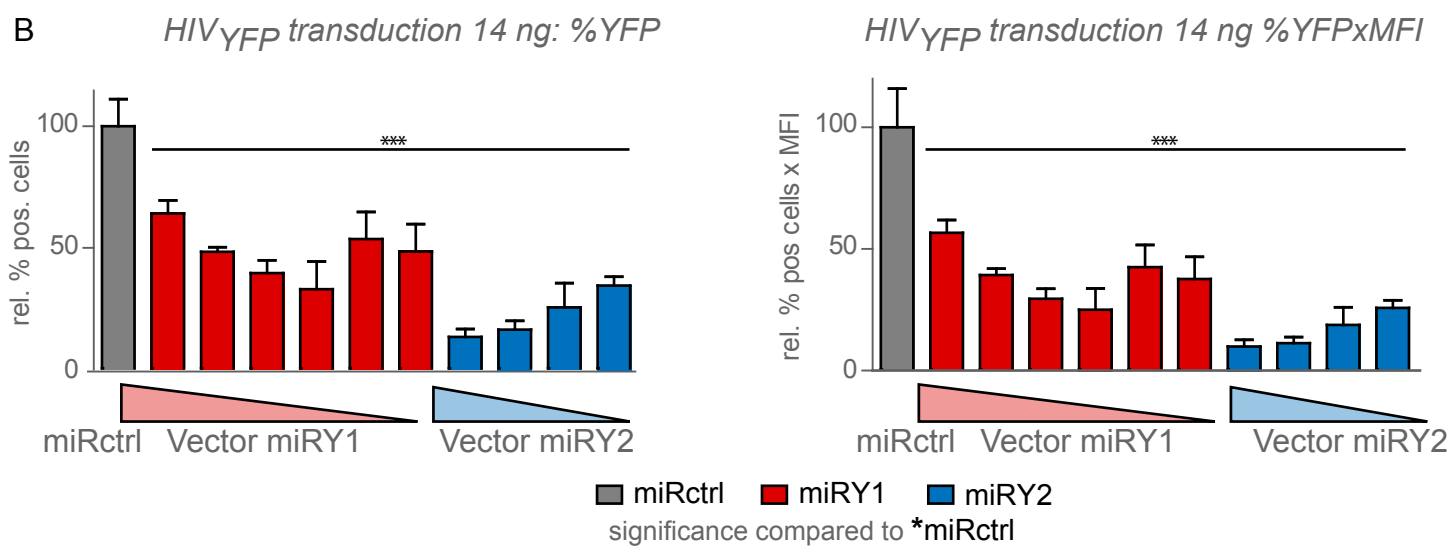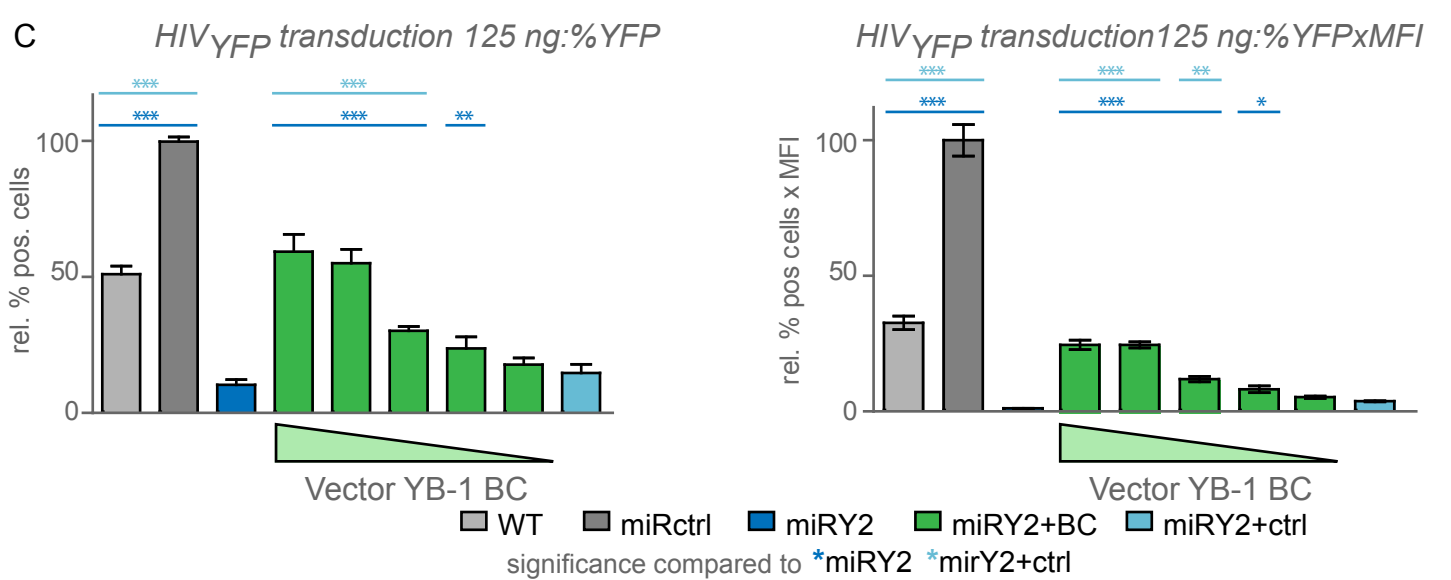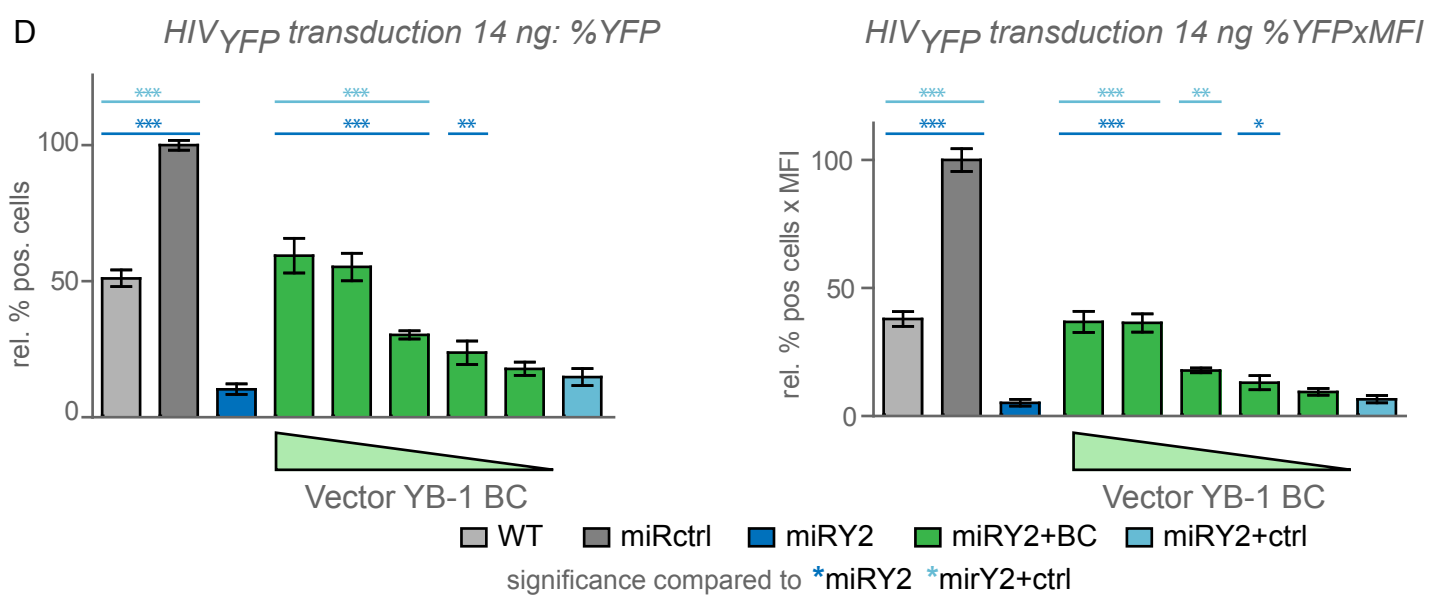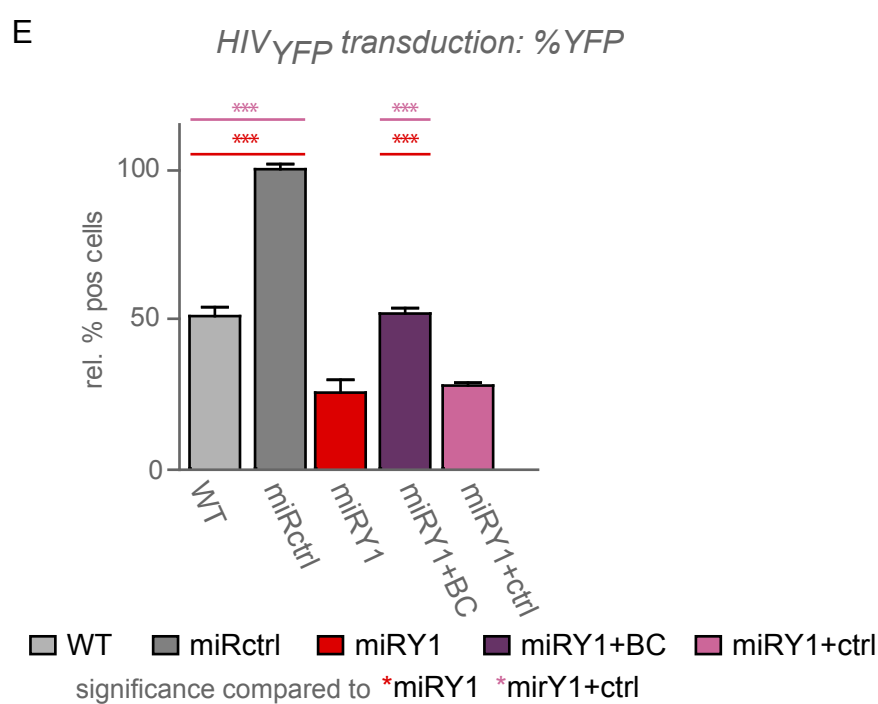

**Fig S3**

Supplement: S5 Fig — (A, B) HeLaP4 cells transduced with a dilution series of vector expressing a miRNA-based shRNA against YB-1 (miRY1, red, miRY2, blue) or miRNA-based shRNA against DSRed (miRctrl, dark grey) were transduced with a dilution series of VSV-G pseudotyped HIVYFP and harvested 48–72 hours post transduction. % YFP positive cells (left panel) and the % YFP positive cells x mean fluorescence intensity (MFI) (right panel) as measured by flow cytometry are depicted. Shown is a representative experiment out of 3 independent experiments. (C, D) HeLaP4 cells with a stable YB-1 knock-down (miRY2) were transduced and selected with a dilution series of vector expressing miR resistant YB-1 (miRY2+BC, green) or control vector (miRY2+ctrl, light blue) prior to infection with different amounts (125 ng p24, C or 14 ng p24, D) of single round VSV-G pseudotyped HIVYFP. Cells were harvested 48–72 hours post transduction. % YFP positive cells (left panel) and the % YFP positive cells x mean fluorescence intensity (MFI) (right panel) as measured by flow cytometry are depicted. (E) Similar results were obtained upon rescue of the YB-1 levels after mirY1 KD (miRY1+BC, violet). A representative experiment out of 2 to 4 experiments is shown. Error bars represent standard deviations of triplicate data points. Statistical differences were determined using one-way ANOVA, followed by the Bonferroni multiple comparison test. Cells were compared to (A, B, C) miRctrl (black stars) and miRY2+BC (green stars), (C-E) miRY1 (red stars), miRY2 (dark blue stars), miRY1+ctrl (pink stars) or miRY2+ctrl (light blue stars) conditions. *p <0.05,**p < 0.01, ***p < 0.001. (PDF) [file pone.0200080.s010.pdf]

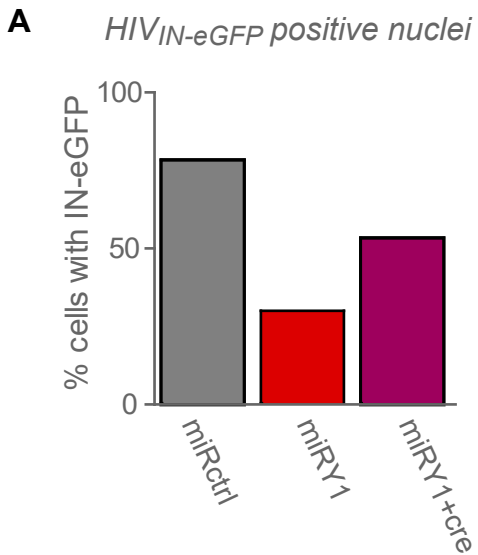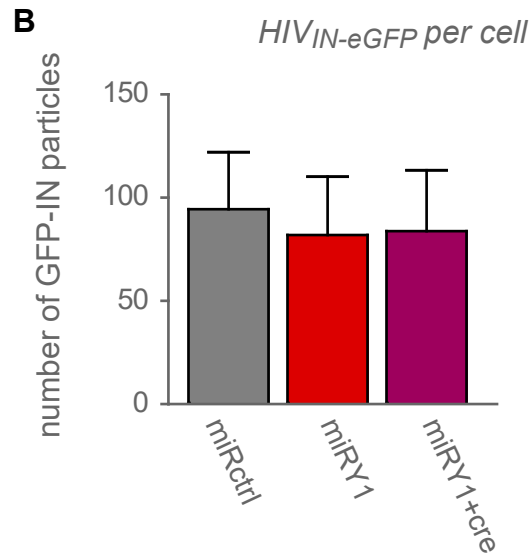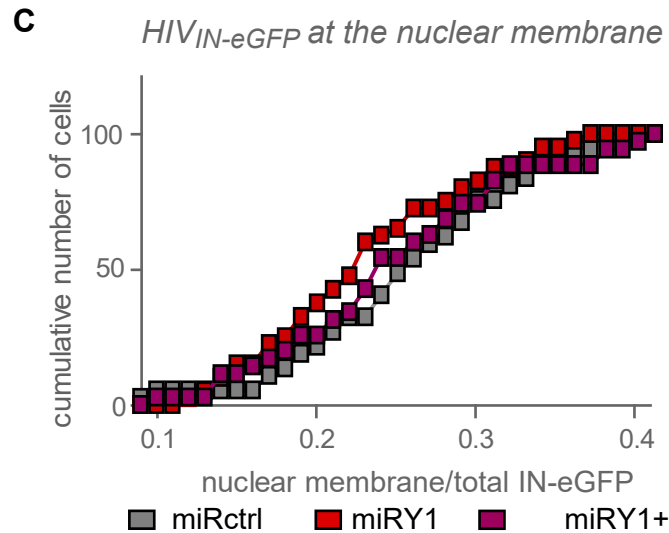

**Fig**

Supplement: S6 Fig — HeLaP4 YB-1 knock-down (miRY1, red, n = 38) or control (miRctrl, dark grey, n = 40) cells were infected with fluorescent HIVIN-eGFP and fixed 5 hours post transduction. As a backcomplementationcontrol the miRY1 was floxed out using Cre–recombinase (miRY1+cre, violet, n = 35). Green fluorescent particles in the cytoplasm and nucleus were quantified by laser-scanning confocal microscopy. The numbers of PICs per (A) cell and at the (B) nuclear membrane are shown. Error bars represent standard deviations. No statistical significant differences between miRctrl, miRY1 and miRY1+cre conditions were observed using (A) one-way ANOVA or (B) Mann-Withney testing. One out of two representative experiments is shown. *p <0.05,**p < 0.01, ***p < 0.001. (PDF) [file pone.0200080.s011.pdf]

*MLV<sub>GFP</sub> transduction: % GFP*

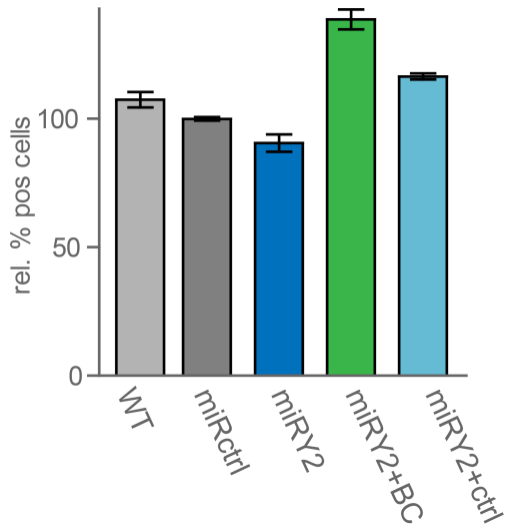

Supplement: S7 Fig — The stable HeLaP4 YB-1 KD cell lines (miRY2) and respective control cell lines were transduced with a VSV-G pseudotyped MLV-vector encoding for eGFP and results were analyzed using flow cytometry. A representative experiment out of 2 experiments is shown. Error bars represent standard deviations of triplicate data points. Statistical differences were determined using one-way ANOVA, followed by the Bonferroni multiple comparison test. Cells were compared to (A, B, C) miRctrl (black stars) and miRY2+BC (green stars), (C-E) miRY1 (red stars), miRY2 (dark blue stars), miRY1+ctrl (pink stars) or miRY2+ctrl (light blue stars) conditions. *p <0.05,**p < 0.01, ***p < 0.001. (PDF) [file pone.0200080.s012.pdf]
